# Strain relaxation in halide perovskites via 2D/3D perovskite heterojunction formation

**DOI:** 10.1126/sciadv.adu3459

**Published:** 2025-06-27

**Authors:** Dongtao Liu, Jinxin Bi, Weidong Xu, Kieran W. P. Orr, Fei Wang, Xueping Liu, Aobo Ren, Jing Zhang, Steven Hinder, Bowei Li, Xiaoguang Luo, Yonglong Shen, Hanlin Hu, Guosheng Shao, Samuel D. Stranks, Lei Su, Wei Zhang

**Affiliations:** ^1^ Advanced Technology Institute, University of Surrey, Guildford GU2 7XH, UK.; ^2^ School of Engineering and Materials Science, Queen Mary University of London, London E1 4NS, UK.; ^3^ Department of Chemical Engineering & Biotechnology, University of Cambridge, Philippa Fawcett Drive, Cambridge CB3 0AS, UK.; ^4^ Department of Physics, Cavendish Laboratory, University of Cambridge, JJ Thomson Avenue, Cambridge CB3 0HE, UK.; ^5^ Department of Materials Science and Engineering, Stanford University, Stanford, CA 94305, USA.; ^6^ Hoffmann Institute of Advanced Materials, Shenzhen Polytechnic University, 7098 Liuxian Boulevard, Shenzhen 518055, China.; ^7^ Institute of Fundamental and Frontier Sciences, University of Electronic Science and Technology of China, Chengdu, 610054, China.; ^8^ The Surface Analysis Laboratory, Department of Mechanical Engineering Sciences, University of Surrey, Guildford, GU2 7XH, UK.; ^9^ Department of Electronics, College of Electronic Information and Optical Engineering, Nankai University, Tianjin 300071, China.; ^10^ State Centre for International Cooperation on Designer Low-Carbon & Environmental Materials (CDLCEM), School of Materials Science and Engineering, Zhengzhou University, Zhengzhou 450001, China.

## Abstract

Applying mechanical strain and strain engineering to halide perovskites has endowed them with intriguing properties. However, an in-depth understanding of mechanical strain, including residual strain in halide perovskites, remains incomplete, coupled with the critical challenge of decoupling strain effects from other interferences. Here, we examine the relaxation of residual tensile strain in three-dimensional (3D) halide perovskites through 2D/3D perovskite heterojunction formation. The 2D perovskite induces structural fragmentation in 3D perovskites, facilitating plastic relaxation of tensile strain. By isolating extrinsic crystalline phase interference and exciton-related optical disturbances, we observe that 3D perovskites retain high crystallinity only with moderate tensile strain relaxation. This moderate relaxation enhances optoelectronic properties in 3D perovskites, including broadened band-to-band absorption and prolonged charge carrier lifetime, markedly contributing to an increase in the maximum attainable power conversion efficiency in photovoltaic devices. Our findings outline conditions for strain relaxation that optimize optoelectronic properties, advancing strain engineering in halide perovskites.

## Teaser

The relaxation of residual tensile strain in 3D halide perovskites is achieved through 2D/3D perovskite heterojunction formation.

## INTRODUCTION

Halide perovskites have been extensively explored due to their impressive properties relevant to photon harvesting and emitting applications (*1*, *2*). Compared to their GaAs and silicon-based equivalents (*3*, *4*), perovskite semiconductors still face pressing challenges, including poor black-phase stability (*5*–*7*), and presence of nonradiative recombination (*8*, *9*), which hampers its realistic deployment in optoelectronic applications. Recent findings suggested that effective use of mechanical strain could be a unique means to further advance the properties of halide perovskites (*10*–*12*). For instance, compressively strained FAPbI_3_ [formamidinum (FA)] has been shown to retain its black-phase properties (*13*) under ambient condition for 1 year without encapsulation (*12*). In addition, compressive strain has been proposed as a contributing factor to the enhancement of carrier mobility in halide perovskites (*12*, *14*). Furthermore, a vertical strain gradient has been demonstrated to induce bandgap gradient in thin-film perovskites (*15*, *16*). All these findings underscore the potential to boost the performance of perovskite optoelectronics using state-of-the-art strain engineering. Despite these encouraging achievements, serious debates persist in elucidating strain effect in halide perovskite.

In fabricating high-performance perovskite optoelectronics, constructing two-dimensional (2D)/3D perovskite heterojunctions by depositing alkylamine ligands onto the surface of 3D perovskites has become the most common approach (*17*–*20*). Early studies indicated that these heterojunctions could regulate enclosed tensile strain through tensile strain relaxation (*21*–*27*) and improve black-phase 3D perovskite stability (*16*, *28*). In these scenarios, tensile strain mainly arises from the thermal expansion mismatch between perovskites and rigid substrates (*15*, *29*–*31*), with phase transitions suggested to contribute to its development, such as phase transition-induced anisotropic texture propagation (*11*, *32*, *33*). However, recent investigations revealed that similar 2D/3D heterojunctions may reduce black-phase 3D perovskite stability and diminish device operational durability (*6*, *7*, *34*, *35*), with interfacial ion migration and charge accumulation proposed as dominant factors in this process, independent of potential strain regulation. Further, these divergent findings may reflect a limited understanding of the mechanisms underlying strain relaxation and accumulation in engineered perovskites, leading to seemingly inconsistent interpretations. Beyond black-phase perovskite stability, the optoelectronic properties of halide perovskites, such as tunable bandgap absorption and emission (*3*) and long charge carrier lifetime (*36*), are crucial for the success of perovskite optoelectronics. In addition, reports associated with the strain regulating optoelectronic properties also vary notably, with tensile-strained perovskites showing both red-shifted (*37*) and blue-shifted photoluminescence (PL) emissions (*12*, *15*). These inconsistencies may be due to the presence of other interferences within strain analysis. For instance, when 3D perovskites are converted into 2D perovskites, the exciton will be generated and cause modulation in the PL characteristics of halide perovskite (*38*), which is beyond the strain modulation. Dissimilar to strain-modulated bandgap energy, exciton-modulating optoelectronic properties occur through the creation of excitonic states lying below the conduction band maximum (*39*–*41*), leading to shifts in PL and absorption onset energy. Given the fact that strain appears to strongly modulate the semiconductor properties of halide perovskites (*16*), there is a critical need within the research community for an in-depth understanding of the mechanisms governing strain variation in engineered perovskites. This understanding once achieved would facilitate the precise isolation of strain-induced effects from extrinsic interferences (*16*), thereby enabling accurate characterization of strain impacts on perovskite properties and informing the development of optimized strain-engineering strategies to enhance halide perovskite performance, particularly in 2D/3D heterojunction configurations.

Here, to examine the optoelectronic properties of halide perovskites exclusively affected by residual strain, the primary objective in this work is to elucidate the mechanisms underlying strain variations induced in 3D perovskites upon modification with 2D perovskites. To this end, we deposit various long-chain alkylamine ligands on top of 3D perovskites. These ligands differ in their ability to form 2D perovskites, enabling detailed observation of potential strain variation. Using grazing incidence wide-angle x-ray scattering (GIWAXS) and x-ray diffraction (XRD), we find that in-plane tensile strain in 3D perovskites relaxes upon forming 2D perovskites on their surface. The most notable relaxation occurs with ligands that strongly induce 2D perovskite formation. This process involves fragmenting 3D perovskite into 2D slabs via the ligand, which alleviates structural bonding between corner-sharing PbI_6_ octahedra and relaxing the in-plane tensile strain plastically. Besides, we observe that radical tensile strain relaxation, linked to phase transition and degradation in 3D perovskites, degrades the optoelectronic properties of 3D perovskites. Optimal optoelectronic properties for 3D perovskites, including broadened bandgap absorption and prolonged carrier lifetime, are achieved only with moderate tensile strain relaxation in 3D perovskites. Further photovoltaic analysis demonstrates a marked increase in power conversion efficiency (PCE), with cells undergoing moderate tensile strain relaxation achieving 25.2%, in contrast to 23.8% observed in control cells.

## RESULTS

### Crystallographic analysis in halide perovskites

**Fig. 1.**
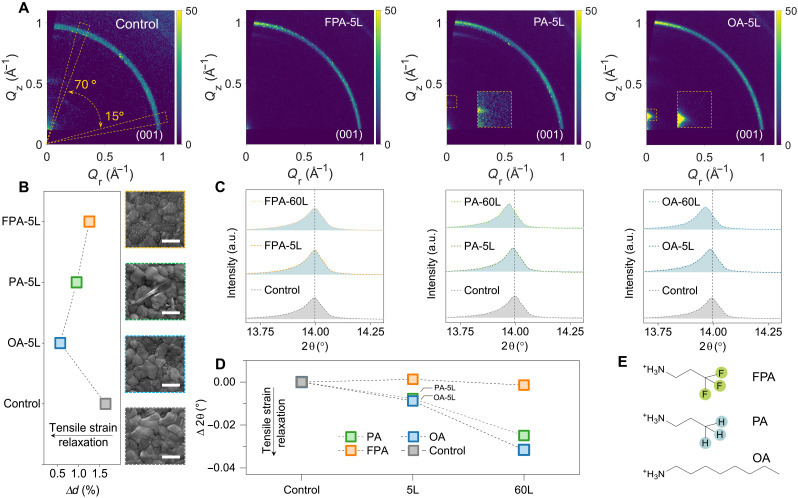
Crystallographic analysis and strain variation in halide perovskites. (**A**) GIWAXS analysis for the control, FPA-5L, PA-5L, and OA-5L. The yellow sector inset the GIWAXS pattern indicates the sector integral of χ = 10° to 15° and χ = 70° to 75°, which is used to extract 1D in-plane and out-of-plane lattice profile. (**B**) Biaxial lattice anisotropy, ∆d , for control and long-chain alkylamine ligand–modified perovskites. The calculation is made using lattice information derived from Fig. 1A. The right panel is the SEM image of the FPA-5L, PA-5L, OA-5L, and the control perovskite from top to bottom. Scale bars, 1 µm. (**C**) XRD patterns for control and long-chain alkylamine ligand–modified perovskites under different annealing conditions. (**D**) Bragg peak shifts at (001) plane between control and long-chain alkylamine ligand–modified perovskites. The 2θ is derived from (C). (**E**) Chemical structure of OA, PA, and FPA. a.u., arbitrary unit.

To initialize the strain analysis, we first carried out crystallographic characterization for the control sample, which is 3D perovskites [(FAPbI_3_)_0.95_(MAPbBr_3_)_0.05_; methylammonium (MA)] deposited on the SnO_2_/ITO (indium tin oxide glass) substrate. For control sample, its x-ray scattering profiles as observed have in-plane *q_xy_* smaller than out-of-plane *q_z_* for (001) plane, suggesting elongated in-plane *d*-spacing (*d_xy_*) with respect to out-of-plane *d*-spacing (*d_z_*), detailed in Fig. 1A and fig. S1A. This observation is characteristic of in-plane tensile strained perovskite crystals (*12*, *29*), consistent with the Poisson effect (*42*). Specifically, for materials with a positive Poisson’s ratio, including halide perovskites, the generation of in-plane tensile strain will result in out-of-plane lattice contraction (*42*), leading to a decrease in *d_z_* relative to *d_xy_*.

By ascertaining the in-plane tensile strain generation in our control sample, we further aim to analyze the potential strain variation for 3D perovskites modified by long-chain alkylamine ligands at their surfaces. Previous evidence suggested that long-chain ligand that has high reaction affinity toward 3D perovskites are prone to convert contacted 3D perovskites into 2D perovskites (*7*). Accordingly, we used three ligands with different reaction affinity towards 3D perovskites to modify their surfaces, including *n*-octylammonium (OA), propylammonium (PA), and 3,3,3-trifluoropropylammonium (FPA) (Fig. 1), Among these ligands, OA with a long alkyl chain, being electron donating, potentially render their NH_3_^+^ group with the highest electron density. Conversely, FPA, characterized by a short alkyl chain and electron-withdrawing fluorine atoms, could exhibit the lowest electron density in the NH_3_^+^ group (detailed in later discussion). Further, with respect to Lewis acids and bases theory (*43*), alkylamine ligands with NH_3_^+^ groups with high electron density, such as OA, will be more likely to react with PbI_6_ octahedra, ideally facilitating the formation of 2D perovskites.

Three modified samples were then subjected to GIWAXS analysis, where the modification is executed by spin-coating different ligands of a relatively low concentration (15 mM) at their surface followed by short-term annealing at 100°C (5 min). The samples here are denoted as OA-5L, PA-5L and FPA-5L, respectively. Considering that the generation of out-of-plane compressive strain (associated with a decrease in *d_z_*) is in response to the in-plane tensile strain (associated with an increase in *d_xy_*) (*16*, *42*), we evaluated the degree of biaxial lattice anisotropy by using $∆d=\frac{d_{\mathrm{xy}}}{d_{z}}-1$ , where *d_xy_* and *d_z_* are derived from GIWAXS patterns (Fig. 1A and fig. S1). Normally, perovskite with higher in-plane tensile strain will deliver a higher ∆d , and a ∆d of zero implies zero strain in the film. This approach is able to assess the intrinsic tensile strain in perovskites without reliance on reference samples. Because of the strong scattering intensity of perovskite (001) plane, we conducted strain analysis by extracting the *d_z_* and *d_xy_* of (001) plane. As shown in Fig. 1B, all three alkylamine ligand–modified samples have a lower ∆d than the control sample ( ∆d ∼ 1.6%). ∆*d* for OA-5L, PA-5L, and FPA-5L is ~0.6, 1.0, and 1.3%, respectively. Meanwhile, the formation of 2D perovskites is confirmed by the distinct scattering spots observed in OA-5L (*q* = 0.235 ${Å}^{-1}$ , *n* = 2) and PA-5L (*q* = 0.345 ${Å}^{-1}$ , *n* = 2) (fig. S2). In comparison, 2D perovskite scattering features are absent in FPA-5L. By monitoring the crystal features among modifications, we noticed that the presence of 2D perovskites in OA-5L and PA-5L correlates closely with the rise of tensile strain relaxation, while FPA-5L, lacking 2D perovskites, shows less strain relaxation. Because GIWAXS probes only the top surface of the perovskite layer (∼200 nm), we corroborated these observations with XRD analysis, which examines the entire thickness of the perovskite layer.

The XRD measurement is performed under Bragg-Brentano geometry, and it evaluates perovskite lattice information in the out-of-plane direction. Considering that Young’s modulus of ITO (116 GPa) (*44*) is an order of magnitude larger than that of perovskites (∼10 to 15 GPa) (*31*) and the compressive stress suffered by the ITO substrate (*16*), and the Bragg peak shift in XRD is negligible compared to the case in strained halide perovskites, we thus use ITO as an internal standard representing strain-free sample. Before strain analysis, all XRD patterns as measured (figs. S3 to S5) were corrected against the diffraction profile of the ITO substrate. In the XRD patterns (Fig. 1, C and D), we noticed that in contrast to the control sample, the Bragg peak of (001) plane in OA-5L and PA-5L samples (2θ ∼ 14°) is shifted toward a lower Bragg angle (defined as a negative shift) by $∆2\theta_{⊥}∼-0.009{}^{\circ}$ ( $∆d_{⊥}∼0.004Å$ ) and $∆2\theta_{⊥}∼-0.008{}^{\circ}$ ( $∆d_{⊥}∼0.003Å$ ), respectively (table S1). Meanwhile, the FPA processes with an inappreciable Bragg peak shift. The negative shift in the Bragg peak in Bragg-Brentano geometry indicates a lengthening of *d*-spacing in the out-of-plane direction and, hence, a reduction in tensile strain at the in-plane direction as per Poisson effect. To further substantiate this strain observation, GIXRD analysis is performed and revealed that the modified perovskites exhibited a comparable reduction in in-plane tensile strain relaxation (fig. S6), consistent with the findings from GIWAXS analysis. In addition, XRD analysis identified negative shifts in the nonreflective (111) plane in OA(PA)-5L samples (figs. S3 and S4), with these shifts being more pronounced. This behavior is characteristic of high Miller index planes in strained halide perovskites, further highlighting the in-plane tensile strain relaxation in the ligand-modified samples.

Considering that our ligand treatment essentially involves a secondary annealing process, we investigated the role of thermal annealing in strain relaxation for the modified samples. This analysis draws upon the well-documented phenomenon of thermal stress relief observed in steel manufacturing (*45*), where controlled heat treatment effectively reduces residual stresses. We began our analysis with FPA-modified samples subjected to an extended thermal annealing duration of 60 min (denoted as 60L). If the thermal stress relief mechanism applies in this context, then we anticipate that the prolonged annealing will render FPA-modified samples with substantially improved tensile strain relaxation. However, further XRD analysis (Fig. 1C) showed that FPA-60L only propagated a marginal negative peak shift against the control samples ( $∆2\theta_{⊥}∼-$0.001°; $∆d_{⊥}∼0.0006Å$ ). Meanwhile, a notably enhanced negative (001) peak shift was observed in OA-60L and PA-60L, with $∆2\theta_{⊥}$ of ∼ −0.032° ( $∆d_{⊥}∼0.014Å$ ) and ∼−0.025° ( $∆d_{⊥}∼0.011Å$ ), respectively. Considering that $∆2\theta_{⊥}$ in OA(PA)-60L remains one-order magnitude larger than that in FPA-60L, the tensile strain relaxation as observed here should not be dominated by the secondary annealing. The enhanced strain relaxation observed in OA(PA)-60L is likely attributed to the increased interaction between ligands in OA(PA)-5L and 3D perovskites. As evidenced in the scanning electron microscopy (SEM) analysis (figs. S7 to S9), OA-60L starts to exhibit the feature of needle-like crystals, and PA-modified samples transit their surface morphology from needle-like crystal-contained surface (PA-5L) to slab-like crystal-contained surface (PA-60L). This dynamic evolution of the surface morphology provides evidence of the ongoing interaction between the ligand and 3D perovskites during prolonged annealing. These interactions are likely to enhance ligand diffusion (*46*) and facilitate the formation of 2D perovskites by converting any residual ligand into 2D structures, thereby promoting tensile strain relaxation. Meanwhile, FPA-60L remains the same surface morphology as FPA-5L in the SEM analysis, indicating the limited reaction affinity of FPA in forming 2D perovskites and thus its poor strain regulation capability. Together, this analysis indicates that tensile strain relaxation is primarily driven by the ligand reaction affinity for forming 2D perovskites, rather than by secondary annealing. Nevertheless, we observed that extended annealing can result in slight phase degradation, as indicated by an increased presence of PbI_2_ in the XRD analysis for all three modified samples (figs. S3 to S5). This degradation would negatively impact the performance of perovskites in optoelectronic applications. Given that tensile strain relaxation has been identified in OA(PA)-5L, we further investigated the underlying mechanisms involved.

### Tensile strain relaxation in halide perovskites

Pioneering works showed that for halide perovskites, phase transition will lead to changes in their enclosed strain (*11*, *32*). However, we do not observe any extrinsic crystallographic texture developed in OA(PA)-5L compared to the control samples (figs. S3 to S5), with all samples predominantly exhibiting cubic phase structures (fig. S10). These findings exclude the possibility of phase transition–induced strain relaxation. Moreover, there is no evidence that OA(PA)-5L underwent phase degradation in its 3D perovskite component during modification, ruling out phase degradation as a cause of strain relaxation. Early investigations into 2D perovskite–modified 3D perovskites suggest that strain relaxation in 3D perovskites is attributed to elastic strain compensation (*30*, *47*) and the buffering effect of contacted 2D perovskites with a lower Young’s modulus (*23*, *24*). However, it should be noted that interlayer strain compensation applies only when related objects are in the form of compact thin films (*30*). In our study, we did not observe 2D perovskites created as compact thin films (figs. S7 to S9), and this morphology is consistent with many other reports (*6*, *48*, *49*). Accordingly, the tensile strain relaxation found in our work cannot be explained by conventional strain compensation. Besides, assuming that tensile strain relaxation in 3D perovskites is facilitated by the buffering effect of 2D perovskites with a low Young’s modulus, FPA and PA, that contains alkyl chain of the same length, ideally should share a similar Young’s modulus (*50*, *51*) and leading to similar regulation on the residual strain in 3D perovskites. However, our observations clearly showed that strain relaxation between these two cases differs notably, with PA-modified samples exhibiting more pronounced strain relaxation (Fig. 1). These observations suggest that elastic deformation including strain compensation and buffering cannot account for the strain relaxation in 3D perovskites, especially when 2D perovskites are created on their surfaces by depositing alkylamine ligands.

**Fig. 2.**
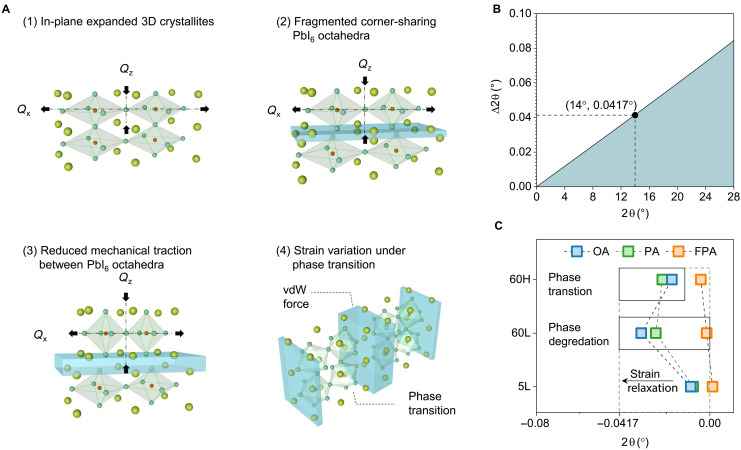
Tensile strain relaxation in halide perovskites. (**A**) Schematic illustration of tensile strain relaxation triggered in 3D halide perovskites. Notably, the lattice plane of 2D perovskites is preferentially aligned in the out-of-plane direction, as evidenced in fig. S2D. (**B**) Calculation of theoretical out-of-plane Bragg peak shifts for perovskites under thermal expansion mismatch-induced strains. (**C**) Summary of the (001) peak shifts in real XRD observation. vdW, van der Waals.

On the other hand, when long-chain alkylamine (monoammonium) ligands are deposited on the top surface of 3D perovskites, 2D perovskites related to Ruddlesden-Popper (RP) phases (*52*) are formed. The formation process of RP 2D perovskites is through the ligand fragmenting the 3D perovskites and converting 3D structure into 2D slabs (*7*, *38*), where the fragmented corner-sharing PbI_6_ octahedra are interlocked by alkylamine ligands via weak van der Waals (vdW) force. Previous evidence showed that in the fabrication of single-crystalline semiconductors, by using the vdW-assisted epitaxy (*53*), the epilayer can be detached from the lattice-mismatched substrate, resulting in suppression of potential strain generation in the epilayer. Likewise, for the polycrystalline halide perovskites deposited onto the rigid substrate (ITO herein), the introduction of vdW bonding between the substrate and perovskites can enable the partial elimination of tensile strain generated in halide perovskites (*54*). Among these reports, one consensus reached is that under the introduction of vdW bond in the system, there is a reduced mechanical bonding between objects and thus a tensile strain relaxation in the system. In our observation, we have determined that tensile strain relaxation, while excluding any elastic deformations, is closely related to the generation of 2D perovskites (Fig. 1), which are enriched with vdW bonding (*38*, *52*). We rationalize our observations as that the alkylamine ligand–triggered fragmentation of 3D perovskites, facilitated by the diffusion of the ligand into the 3D perovskite lattice, leads to the breaking of covalent bonds between corner-sharing PbI_6_ octahedra. This is accompanied by the formation of weaker vdW interactions, which reduce interlayer cohesion and allow each corner sharing layer to relax more easily, reducing its tensile strain, as depicted in Fig. 2A. Given the irreversible nature of 3D perovskite fragmentation, the associated tensile strain relaxation can be categorized as plastic deformation.

Furthermore, we investigated the strain heterogeneity along the perovskite thickness and its correlation with surface treatment. Our findings indicate that strain tends to accumulate more prominently at the surface regions, where the perovskite exhibits high surface compactness and crystal integrity. Moreover, the effectiveness of ligand treatment is strongly influenced by the intrinsic compactness of the perovskite film. For example, surface treatment–induced strain modulation is substantially more evident in perovskites with high film compactness, corresponding to the surface regions of halide perovskites (note S1 and figs. S11 and S12).

Motivated by that tensile strain relaxation as confirmed is associated with negative Bragg peak shifts, we further quantified the extent to which Bragg peak shifts indicate complete relaxation of tensile strain induced by thermal expansion mismatch. In addition, we assessed the feasibility of tensile strain fully relaxed in halide perovskites. Considering thin-film perovskites deposited on a rigid substrate cooling from 100° to 25°C, the tensile strain and compressive strain are generated in perovskites at respectively in-plane and out-of-plane directions (*30*, *31*). In association with the calculation of theoretical out-of-plane compressive strain (note S2), the Bragg peak shift for a specific plane under this strain can be ascertained from Eq. 1 (*55*)

ε=−∆θcotθ (1)

For the (001) plane, 2θ ∼ 14°, the theoretical out-of-plane compressive strain will cause a Bragg peak shift of ~0.0417° (Fig. 2B and fig. S13). Considering the relaxation process of this strain, the corresponding negative Bragg peak shifts will be no more than ∼−0.0417°. Recalling the XRD analysis, it can be found that the perovskite of PA-5L and OA-5L has their $∆2\theta_{⊥}$ less than ∼−0.0417° (Fig. 2C), which highlights in-plane tensile strain only partially relaxed in their structure and consistent with GIWAXS analysis.

Because tensile strain relaxation is aligned with the formation of 2D perovskites, we assess to fully relax the tensile strain by increasing the amount of 2D perovskites created in 3D perovskites. To this end, we used alkylamine ligand of high precursor concentration (200 mM) to modify the 3D perovskite surface, followed by thermal annealing at 100°C for 60 min (denoted as 60H). However, contrary to our expectations, a slight α-to-β phase transition was induced in OA(PA)-60H (note S3 and fig. S14), leading to an unexpected increase in tensile strain relative to OA(PA)-60L. Notably, compared to the control sample, the tensile strain remained reduced in OA(PA)-60H (Fig. 2C). For a clear demonstration, we regarded the tensile strain relaxation without phase degradation and transition as moderate tensile strain relaxation, as seen in OA(PA)-5L. Conversely, tensile strain relaxation that is involved with phase transition and degradation is considered radical tensile strain relaxation, as seen in OA(PA)-60L and OA(PA)-60H. Although pioneering work suggested that tensile strain relaxation can promote the optical properties of halide perovskites (*16*), the radical strain relaxation observed may not be necessarily linked to better optical properties (*16*, *56*). As optical property is crucial for the success of the perovskite optoelectronics, we further thoroughly analyzed the strain effect on the optical properties of halide perovskites.

### Optical properties in strain-relaxed halide perovskites

**Fig. 3.**
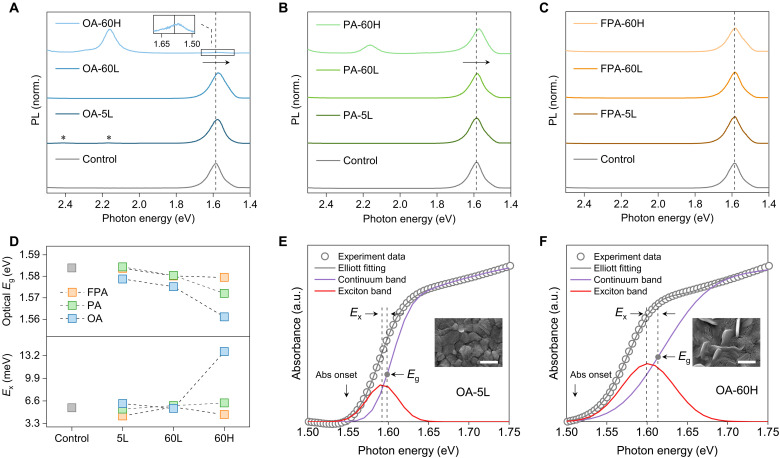
Optical analysis for strain-relaxed halide perovskites. Steady-state PL measurement for (**A**) OA-, (**B**) PA-, and (**C**) FPA-modified samples. The PbI_2_ emission was invisible during the PL investigation. The asterisk mark in (A) denotes the OA-associated 2D perovskites. (**D**) Optical bandgap energy and exciton binding energy for the control and modified perovskites. Elliott modeling of absorption spectra for (**E**) OA-5L and (**F**) OA-60H. The purple curve represents the band-to-band absorption, and the red peak represents the exciton-related absorption. The *E*_x_ and *E*_g_ denote the exciton binding energy and bandgap energy, respectively. Inset: SEM images of the corresponding perovskite films. Scale bars, 1 μm.

In halide perovskite optoelectronics, two crucial optical properties of perovskites that substantially influence device performance are the bandgap emissions (or absorption) and the charge carrier lifetime. We first analyzed the modified perovskites under different strain conditions in the steady-state PL measurement. In general, a shift in PL emissions reflects the changes in the bandgap energy of halide perovskites (*16*). In the PL spectra (Fig. 3, A to C), an emission peak at ∼1.58 eV was assigned to 3D perovskites. In strain-relaxed perovskites, a red shift of this PL emission peak is observed compared to the control sample. For instance, a notable PL red shift of Δ*E* = 9 meV was detected for OA-5L. In contrast, this PL shift is negligible for FPA-5L (Fig. 3C), consistent with its less pronounced tensile strain relaxation. With the increased tensile strain relaxation in OA(PA)-60L (Fig. 1), these perovskites exhibited greater PL red shifts compared to OA(PA)-5L (Fig. 3, A and B). However, a reduced absorption coefficient was also noted for OA(PA)-60L (fig. S15), which could be attributed to phase degradation associated with radical tensile strain relaxation. This analysis demonstrated that radical tensile strain relaxation can be detrimental for halide perovskites.

Because of the active formation of 2D perovskites in OA(PA)-5L (Fig. 1A), we further access the potential exciton resonances on the regulation of optical bandgap properties (*39*, *40*, *57*). By running the Elliott modeling (figs. S16 to S18) to the absorption spectra, the exciton binding energy (*E*_x_) can be extracted for samples (Fig. 3D and table S2). For OA(PA)-5L and FPA-5L, the *E*_x_ as determined remains nearly constant with value less than 6.2 meV (Fig. 3D) and it fell in the normal range for 3D perovskites (*58*). Consequently, any PL red shifts observed in OA(PA)-5L (Fig. 3, A and B) should be dominated by the tensile strain relaxation rather than exciton resonance. In XRD analysis, our strain observations appear to follow Poisson effect where the relaxation of out-of-plane compressive strain is affiliated with the relaxation of in-plane tensile strain (Fig. 1). However, our PL red shifts are likely a response solely to the relaxation of in-plane tensile strain, as the tensile strain relaxation is reported to trigger the upward movement of valence band maximum and thus the shrinkage of bandgap structures (*12*, *15*). In contrast to the samples here that suffered from biaxial strain (*12*), the previous report (*37*) about blue-shifted PL emission in perovskites is typically under the regulation of uniaxial compressive strain, without specifying whether a phase transition occurred in these perovskites. The notable difference in strain-regulated PL shifts underscores the importance of identifying the strain type before analyzing its effects on halide perovskites.

Although we have subjected strain variation observed in OA(PA)-60H as radical tensile strain relaxation, where the α-to-β phase transition as triggered leads to reduced absorption coefficients for OA(PA)-60H within the range of 1.7 eV to 2.4 eV (fig. S15), it is still crucial to investigate their strain-related optical bandgap properties. As observed in Fig. 3 (A and B), OA(PA)-60H exhibited a slight red shift in PL compared to OA(PA)-60L. This red shift is further corroborated by the observed variations in optical bandgap energy (Fig. 3D), which is determined through *T*_auc_ plot analysis. Nevertheless, our XRD analysis does not support that OA(PA)-60H maintains a reduced tensile strain relative to OA(PA)-60L (note S3). Meanwhile, a pronounced increase in the *E*_x_ is observed for OA(PA)-60H, with OA-60H reaching 13.8 meV (Fig. 3D) and PA-60H exhibiting the highest *E*_x_ of 6.3 meV relative to PA-60L and PA-5L. This enhanced *E*_x_ is accompanied by the emergence of an intense PL peak associated with 2D perovskites, centered around ∼2.16 eV (Fig. 3, A and B). Together, the unexpected red shifts in PL and optical bandgap energy in OA(PA)-60H could be interpreted by a synergistic effect of strain and enhanced exciton resonance in the 2D/3D perovskites, as exemplified in Fig. 3 (E and F). Under enhanced exciton resonance, the formation of excitonic states below the conduction band minimum in perovskites substantially contributes to the red shift observed in both PL and optical bandgap energy. One similar report is that for strain-relaxed perovskites, the observed PL red shift is due to the creation of delocalized states at the band tail (*41*, *59*). As PL characterization is also used to indirectly assess strain in halide perovskites, the practice here indicates that a red shift in its optical bandgap does not inherently suggest tensile strain relaxation. Instead, the observed red shift in the optical bandgap could be primarily attributed to other optical disturbances, such as increased exciton resonance. This insight can inform the selection of optimal strain-engineered perovskites for improved device designs.

In addition to the radical tensile strain relaxation that reduces the absorption coefficient in halide perovskites, moderate tensile strain relaxation, as exemplified in OA-5L, optimizes optical properties. This optimization is evidenced by a broadened band-to-band absorption and a preserved absorption coefficient following moderate strain relaxation in the perovskites. Further, we analyzed the charge carrier characteristics for OA-5L, PA-5L, and FPA-5L upon moderate tensile strain relaxation.

### Photoluminescence carrier lifetime in strain-relaxed halide perovskites

**Fig. 4.**
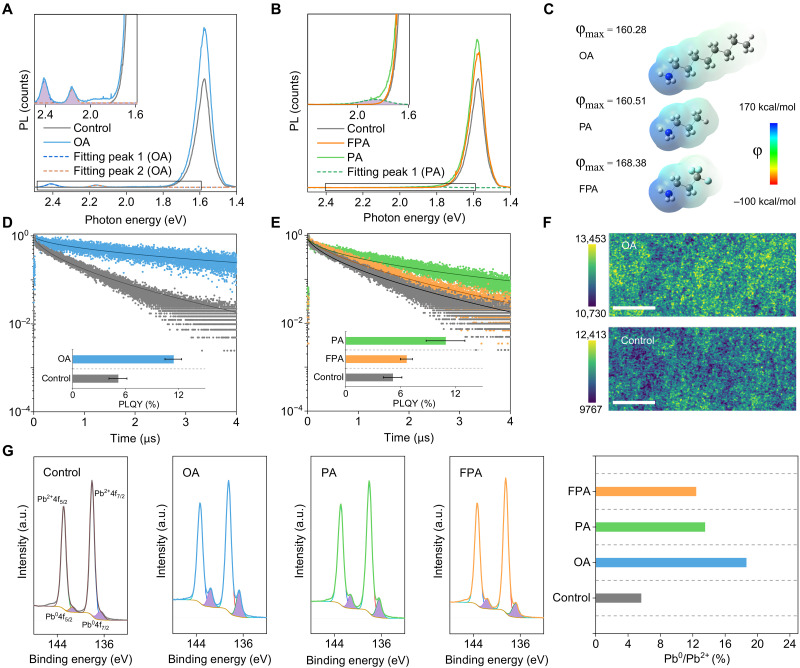
Photoluminescence analysis for strain-relaxed halide perovskites. Steady-state PL measurement for (**A**) control and OA-5L, (**B**) PA-5L, and FPA-5L. The inset enlarged spectra are to highlight the formation of 2D perovskites in OA-5L and PA-5L. (**C**) Electrostatic potential mapping for OA, PA, and FPA, respectively. The decrease of electrostatic potential (φ) indicates the increase in electron density. TRPL spectra probed at ~1.58 eV for (**D**) control and OA-5L, (**E**) PA-5L, and FPA-5L on glass substrate. Inset: PLQY measurement for corresponding perovskites. Error bars: SD. *n* = 3. (**F**) Broadband PL mapping for control and OA-5L on glass substrate. Scale bars, 50 μm. A continuous-wave 405-nm laser with intensity of 82.4 mW/cm^2^ equivalent to 1 sun was used here for excitation. (**G**) Pb 4f XPS measurement and derived peak area ratios of Pb^0^ to Pb^2+^ in control, OA-5L, PA-5L, and FPA-5L.

Before time-resolved PL (TRPL) measurements to examine carrier lifetimes in halide perovskites, a notable increase in steady-state PL intensity at 1.58 eV was observed in three modified perovskites relative to controls (Fig. 4, A and B). OA-5L displayed the greatest PL intensity enhancement, while FPA-5L showed the least. This enhancement appears linked to tensile strain relaxation, with OA-5L showing both the highest PL intensity and lowest tensile strain. Besides, the 2D perovskite peaks have been determined at 2.41 eV (*n* = 1) and 2.17 eV (*n* = 2) for OA-5L (Fig. 4A) (*52*). Likewise, PA-5L displayed a shoulder at 1.86 eV (Fig. 4B), absent in FPA-5L. The lack of 2D features in FPA-5L correlates with its lower NH_3_^+^ electron density, derived from the density functional theory calculation (Fig. 4C), resulting in minimal strain relaxation and limited PL modulation in 3D perovskites.

To clarify the link between strain relaxation and PL quenching pathway, TRPL analysis is conducted. Among samples, OA-5L sample achieved the longest carrier lifetime (2156 ns) (Fig. 4, D and E, and table S3), surpassing PA-5L and FPA-5L with lifetimes of 723.8 and 553.3 ns, respectively. This result is further corroborated by PL quantum yield (PLQY) measurement, with the OA-5L reaching the highest PLQY at 11.4% (Fig. 4, D and E). Besides, the uniform PLQY enhancement across modified samples suggests that the potential formation of local 2D/3D heterojunction does not modify the intrinsic photoinduced carrier transport in 3D perovskites, which otherwise lowers PLQY by inhibiting radiative recombination. Moreover, the consistent *E*_x_ across modified samples, as confirmed by Elliott modeling, indicates that PLQY improvements here are not due to radiative excitonic recombination (*60*). Together, it deduced that enhanced PL properties in modified samples originate from suppressed nonradiative recombination pathways. The increase in carrier lifetime (Fig. 4, D and E) aligns with steady-state PL results, pointing to in-plane tensile strain relaxation as a key factor in reducing nonradiative recombination in halide perovskites.

Considering that commonly used XRD systems (including our own) feature a beam footprint ranging from a few square millimeters to centimeters (*61*), which is several orders of magnitude larger than the perovskite grain size (*62*), the residual stress probed in this study belongs to type I residual stress (*45*, *63*). Type I residual strain reflects the macroscopic lattice distortion of multiple crystallites (*45*). To evaluate macroscopic PL in strain-relaxed samples, PL mapping was conducted on the OA-5L sample under 1-sun equivalent laser fluence. As shown in Fig. 4F, the PL intensity in OA-5L is uniformly increased compared to the control sample (strain-rich), further confirming that tensile strain relaxation reduces nonradiative recombination across the film. Moreover, by applying ligand modification toward wide-bandgap (*E*_g_ = ∼1.68 eV) and low-bandgap perovskites (*E*_g_ = ∼1.53 to 1.55 eV) (*64*), similar PL modulation for perovskites under moderate strain relaxation is observed including PL red shifts and enhanced steady-state PL intensity, confirming the universality of our finding (fig. S19).

Besides, we observed through x-ray photoelectron spectroscopy (XPS) analysis (Fig. 4G and fig. S20) that modified perovskites showed a slight increase in Pb^0^ peak intensity compared to the control, with the OA-5L displaying the highest Pb^0^ peak intensity among the three modifications. However, the XRD analysis does not reveal any notable Pb^0^ peaks in the modified perovskites (fig. S21), indicating that Pb^0^ formation is minimal and restricted to trace levels. Together, this analysis implies that the trace amount of Pb^0^ formation plays a secondary role in the modulation of nonradiative recombination. Instead, the suppressed nonradiative recombination here is potentially related to minimized recombination centers upon tensile strain relaxation, as in-plane tensile strain relaxation is generally associated with the reduction of biaxial lattice distortion that potentially contributes to the decrease of structural defects and recombination centers in halide perovskites (*15*, *63*, *65*, *66*). Nevertheless, it is imperative to recognize that a substantial increase in Pb^0^ can induce detrimental effects, as evidenced by reports demonstrating a marked degradation of both device performance and long-term stability (*46*), particularly under conditions of thermal and light stimulation.

### Photovoltaic properties in strain-relaxed halide perovskites

**Fig. 5.**
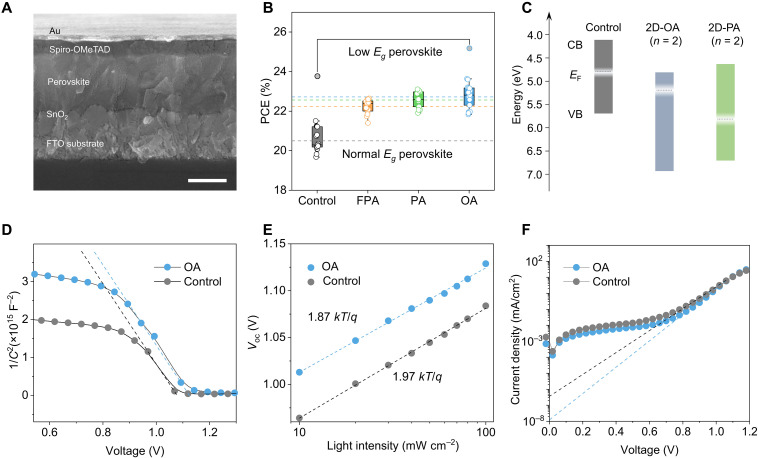
Photovoltaic analysis for strain-relaxed halide perovskites. (**A**) Cross-sectional SEM for a representative solar cell. Scale bar, 500 nm. (**B**) PCE statistics for perovskite solar cells (0.09cm^2^) with and without modifications. The dashed line represents the average PCE for each group using normal bandgap perovskites, *E*_g_ ~ 1.58 eV, *n* = 16. The circular data points shaded in gray represent PCE attained in solar cells using low bandgap perovskites, *E*_g_ ~ 1.55 eV. For the modified solar cells, the perovskite layers used are OA-5L, PA-5L, and FPA-5L, respectively. (**C**) UPS spectra and absorption spectra revealed the Fermi level (*E*_F_) position, conduction band (CB), and valance band (VB) of control 3D perovskite and (OA)_2_(FA)Pb_2_X_7_ and (PA)_2_(FA)Pb_2_X_7_. (**D**) Mott-Shockey plots for control and OA-modified perovskite solar cells in the dark. (**E**) Light intensity–dependent *V*_oc_ measurement for the control and OA-modified perovskite solar cells. (**F**) Dark *J*-*V* curves for the control and OA-modified perovskite solar cells. The medium bias range is used to determine the dark saturation current, mitigating the effects of series and shunt resistance.

To investigate whether strain-relaxed perovskite thin-film conveys promising photovoltaic performance, the n-i-p–structured solar cells (FTO/SnO_2_/alkylamine modified perovskite/Spiro-OMeTAD/Au) as a conceptual demonstrator were fabricated and compared, and a typical cross section SEM image for the control solar cell is shown in Fig. 5A. The *J*-*V* results showed that the modified perovskite devices exhibited substantially increased PCE in average compared to the control, and OA-modified devices preserved the highest PCE among three groups (Fig. 5B). This optimal PCE obtained in OA-modified solar cells is highly consistent to previous optical analysis, in which strain-relaxed OA-modified perovskites preserved the most pronounced bandgap shrinkage and suppressed nonradiative recombination among modifications. The OA-modified devices, incorporating an absorber with *E*_g_ ∼ 1.58 eV (referred to as normal bandgap perovskite), demonstrate an enhancement in maximum attainable PCE, achieving 23.6% compared to 21.5% in the control devices (fig. S22A and table S4). This improvement is corroborated by maximum power point (MPP) tracking measurements (fig. S22B). Furthermore, when a low-bandgap perovskite (*E*_g_ ∼ 1.55 eV) is used as the absorber, OA-modified devices achieve an impressive PCE of 25.2%, surpassing the control devices, which exhibit a PCE of 23.8% (fig. S23A). Across these devices, the discrepancy in short-circuit current density (*J*_sc_) between *J*-*V* and external quantum efficiency (EQE) measurements remains below 3% (fig. S23B).

We noticed that the substantial PCE enhancement in OA-modified devices is primarily caused by an increase in open-circuit voltage (*V*_oc_) (table S4). To elucidate the potential impact of energy band alignment induced by surface treatments, ultraviolet photoelectron spectroscopy (UPS) measurements were conducted, complemented by XPS analysis. Initially, the 2D perovskite layers were treated as discrete entities, and their band structures were evaluated independently (fig. S24). The analysis reveals that the OA- and PA-based 2D perovskites with an *n* = 2 configuration (fig. S2) may act as barriers to efficient hole extraction between 3D perovskites and Spiro-OMeTAD (Fig. 5C). An alternative framework was also considered, wherein the 3D perovskite constitutes the main entities for analysis and long-chain ligands function as electronic modifiers (*6*, *7*, *67*). Nevertheless, similar effects were observed (figs. S20C and S25). The resulting band bending at the interface can lead to enhanced recombination losses, typically manifesting as a reduction in *V*_oc_ and overall device performance (*68*). Contrary to this expectation, our experimental results demonstrate an increase in *V*_oc_ and improved device efficiency following surface treatment. These findings indicate that the *V*_oc_ enhancement in OA-modified devices cannot be attributed to surface treatment induced band alignment.

Generally, the *V*_oc_ is highly associated with the built-in potential of solar cells. The improved band alignment at the junction (*68*) or increased photocarrier density in perovskites (*69*) can result in increased built-in potential (*V*_bi_). We identified the *V*_bi_ for solar cells by running the Mott-Schottky analysis (Fig. 5D). The result showed the *V*_bi_ for OA-modified devices was notably higher than that of control solar cells (1.12 V versus 1.07 V). Given that interface band alignment does not primarily drive the observed enhancement in *V*_oc_, it can be inferred that the improvements in *V*_bi_ and *V*_oc_ are predominantly due to the relaxation of tensile strain. This relaxation effectively suppresses nonradiative recombination within perovskite material, thereby facilitating the efficient collection of photocarriers within devices. Light intensity–dependent *V*_oc_ measurements (Fig. 5E) revealed a reduced linear fit slope in OA-modified device compared to control (1.87 kT/q versus 1.97 kT/q), suggesting reduced nonradiative recombination losses. Similarly, the strain-relaxed OA devices exhibit a reduced current leakage as viewed in Fig. 5F. The reverse saturation current in OA devices is decreased to 1.71 × 10^−8^ mA/cm^2^ compared to the control devices of 4.96 × 10^−7^ mA/cm^2^, representative of suppressed unwanted carrier transport crossing *P*-*N* junction under dark (*70*). To strengthen this conclusion, a half-stack device with control and OA-5L samples coated with Spiro-MeOTAD was prepared. Under 1 sun laser excitation, PL measurements revealed notably enhanced PL intensity in OA-5L sample (fig. S26), indicating that ligand treatment promotes radiative recombination and is expected to enhance quasi-Fermi level splitting and *V*_oc_ in devices (*71*).

We investigated that moderate strain relaxation enhances perovskite optical properties, including broadening band-to-band absorption and extending charge carrier lifetimes, thereby advancing the maximum attainable PCE in solar cells. Conversely, radical tensile strain affiliated with phase transition and degradation rather degrades these optical properties. The stability analysis (fig. S27) indicates a similar trade-off between radical strain relaxation and device stability. In dark storage conditions, OA-modified devices demonstrated optimal stability among devices, exhibiting less than 5% PCE decay after 1000 hours of aging. However, under light exposure, the stability of OA-modified devices was surpassed by FPA-modified devices, a trend that became even more pronounced under thermal aging conditions (fig. S27C). This unusual trend is unlikely to result from the hygroscopic nature of perovskite surface, as OA-modified perovskites exhibit greater dewetting in response to water exposure (fig. S28) than FPA-modified counterparts. Conversely, as we confirmed that OA-modified perovskites, under prolonged thermal annealing, exhibited a diminished absorption coefficient associated with radical tensile strain relaxation, this comparably reduced stability in OA-modified devices is more likely to be attributed to radical tensile strain relaxation, potentially coupled with an increase in the detrimental Pb^0^ component (*46*). Thermal annealing and radiative heating (during light exposure) appear to accelerate this strain relaxation process, which in turn compromises device stability. Hence, from a strain engineering perspective in halide perovskites, we propose that, moving forward, incorporating a blend of multiple long-chain alkylamine ligands with varying reactivity should be pursued. This strategy is expected to maximize device performance by facilitating moderate tensile strain relaxation while concurrently preserving device stability under demanding operational conditions.

## DISCUSSION

In summary, we investigated that the construction of 2D/3D perovskite heterojunction can considerably relax the residual tensile strain in 3D perovskites. During the creation of 2D perovskites, corner-sharing PbI_6_ octahedra in 3D perovskites are fragmented by long-chain alkylamine ligand, which diminishes intercrystalline traction and facilitates the relaxation of in-plane tensile strain. This mechanism is identified under the regulation of plastic deformation, differing from the conventional understanding that elastic deformation dominates this process. Moreover, contrary to the general conception, our study reveals that radical tensile strain in 3D perovskites, associated with phase transition and degradation, does not inherently enhance the optoelectronic properties of 3D halide perovskites. While sustaining a high optical absorption coefficient, 3D perovskites exhibit broadened band-to-band absorption and extended charge carrier lifetime only under moderate tensile strain relaxation, a critical factor for maintaining high crystallinity in halide perovskites. This further supports the improvement in the maximum PCE attainable in photovoltaic cells. Moreover, as similar strain variations are observed in ligand-modified perovskites with different bandgaps, our practices provide insights for advancing strain engineering in halide perovskites via interfacial modification, particularly with selected modifiers that induce substantial recrystallization and/or structural fragmentation within adjacent 3D perovskite layers, therefore facilitating the development of mechanically toughened perovskite electronics with superior performance.

## MATERIALS AND METHODS

### Materials

All commercial available reagents were used as received without further purification, including dimethylformamide (DMF; Sigma-Aldrich), dimethyl sulfoxide (DMSO; Sigma-Aldrich), formamidinium iodide (FAI; Greatcell Solar), methylammonium bromide (MABr; Greatcell Solar), methylammonium iodide (MAI; Greatcell Solar) cesium iodide (CsI; Sigma-Aldrich), lead iodide (PbI_2_; TCI), lead bromide (PbBr_2_; TCI), methylammonium chloride (MACl; Sigma-Aldrich), tin (II) chloride dihydrate (SnCl_2_·H_2_O; ≥99.995%, Sigma Aldrich), SnO_2_ colloid precursor [tin (IV) oxide, 15% in H_2_O colloidal dispersion, Alfa Aesar], thioglycolic acid (TGA; Sigma-Aldrich), urea (Alfa Aesar), 2,2′,7,7′-tetrakis[*N*,*N*-di (4-methoxyphenyl)amino]-9,9′-spirobifluorene (Spiro-OMeTAD, Xi’an Polymer), 4-*tert*-butylpyridine (Sigma-Aldrich), bis-(trifluoromethane)sulfonimide lithium salt (LiTFSI; 99.95%, Sigma-Aldrich), FK209 Co(III) TFSI salt (Sigma-Aldrich), acetonitrile (ACN; Sigma-Aldrich), isopropyl alcohol (IPA; Sigma-Aldrich), chlorobenzene (CB; anhydrous, 99.8%, Sigma-Aldrich), 3,3,3-Trifluoro-1-propanamine hydrochloride (FPA; Sigma-Aldrich), propylamine hydrochloride (PA; Sigma-Aldrich), and *n*-octylammonium iodide (OA; Sigma-Aldrich).

### Thin-film and device fabrication

The SnO_2_/ITO (indium tin oxide) substrate was prepared by spin-coating SnO_2_ solution [2.67 weight %, diluted by deionized (DI) water] onto ultraviolet-ozone (UVO)-cleaned ITO substrate (10 min), followed by thermal annealing of the substrate at 150°C for 30 min. The SnO_2_/FTO (fluorine-doped tin oxide) substrate was prepared using a reported chemical bath deposition (CBD) (*72*).

The normal-bandgap 1.4 M (FAPbI_3_)_0.95_(MAPbBr_3_)_0.05_ perovskite precursor solution was prepared by mixing 1.4 M FAPbI_3_, 0.7 M MAPbBr_3_, 0.5 M MACl, and an additional 5 mol % excess of PbI_2_ in a mixed solvent (DMSO/DMF = 1:8). The precursor solution was spin-coated onto various substrates at 1000 rpm for 10 s and at 5000 rpm for the 30 s, where antisolvent quenching (200 μl of CB) was applied onto the rotating film 20 s before end of the spinning. The resultant film was annealed at 100°C for 1 hour in an N_2_-filled glove box.

The wide-bandgap 1.4 M Cs_0.05_FA_0.8_MA_0.15_Pb(I_0.75_Br_0.25_)_3_ perovskite precursor solution was prepared by mixing 1.12 M FAI, 0.21 M MABr, 0.98 M PbI_2_, 0.42 M PbBr_2_, and 0.07 M CsI in a mixed solvent (DMSO/DMF = 1:4). The precursor solution was spin-coated onto the substrates at 1000 rpm for 10 s and at 5000 rpm for the 30 s, where antisolvent quenching (200 μl of CB) was applied onto the rotating film 10 s before end of the spinning. The resultant film was annealed at 100°C for 1 hour in an N_2_-filled glove box.

The low-bandgap 1.4 M Cs_0.05_MA_0.1_FA_0.85_PbI_3_ perovskite precursor solution was prepared by mixing 0.07 M CsI, 0.14 M MAI, 1.19 M FAI, 1.4 M PbI_2_, 0.21 M MACl, and an additional excess of 5 mol % PbI_2_ in a mixed solvent (DMSO/DMF = 155:745). Notably, for application in solar cells, the precursor concentration was proportionally increased to 1.67 M. The precursor solution was spin-coated onto the substrates at 1000 rpm for 10 s and at 5000 rpm for the 35 s, where antisolvent quenching (200 μl of CB) was applied onto the rotating film 15 s before end of the spinning. The resultant film was annealed at 120°C for 10 min in an N_2_-filled glove box.

The 2D perovskites are prepared with a composition of (LA)_2_(FA)_(*n*−1)_Pb_n_X_(3*n*+1)_, where LA represents the long chain ligand, including OA and PA. One molar perovskites solution for each group (*n* = 1 and *n* = 2, respectively) is prepared in a mixed solvent (DMSO/DMF = 1:8). The precursor solution was spin-coated onto a substrate at 1000 rpm for 10 s and at 5000 rpm for the 30 s. The resultant film was annealed at 100°C for 10 min in an N_2_-filled glove box.

To investigate the strain effect, long-chain alkylamine ligands of different concentrations (15 and 200 mM, respectively) in IPA were spin-coated onto the spinning 3D perovskite substrate at 3000 rpm for 30 s, followed by thermal annealing under 100°C of different time (5 and 60 min, respectively) in N_2_-filled glove box.

For the solar cell application, 2D perovskite capping layers were fabricated by spin-coating 15 mM long-chain alkylamine ligand in IPA onto the spinning 3D perovskite substrate at 3000 rpm for 30 s, followed by thermal annealing under 100°C of 5 min in N_2_-filled glove box.

The solar cells were fabricated by spin coating the 70 μl of Spiro-OMeTAD solution onto the perovskite/SnO_2_/FTO substrate at 4000 rpm for 30 s. The precursor solution was made by mixing 100 mg of Spiro-OMeTAD, 39 μl of tBP, 23 μl of Li-TFSI (1.8 M in ACN), and 10 μl of FK209 (0.25 M in ACN) into 1094 μl of CB. Last, the 100-nm Au electrode was prepared by thermally evaporating onto the Spiro-OMeTAD layer (evaporation rate: 0.3 Å/s).

### Characterizations

SEM characterization was taken with JEOL JSM-7100F SEM. An acceleration voltage of 15 kV was applied during the observation. The samples were sputtered by 6-nm Au for better conductivity.

The XPS was recorded using ThermoFisher Scientific Instruments (East Grinstead, UK) K-Alpha+ Spectrometer, equipped with a monochromated Al Kα x-ray source (*h*ν = 1486.6 eV). High-resolution core-level electron spectra were acquired with a pass energy of 50 eV. All spectra acquired were charge referenced against C 1s peak at 285 eV to exclude any charging effects. UPS measurements were conducted using a helium discharge lamp (*h*ν = 21.22 eV) as the excitation source, with a −9 V bias applied between the samples and the detectors.

XRD and GIXRD analysis was conducted using a PANalytical X’Pert Pro powder diffractometer equipped with a Cu Kα target at 45 kV, 1.54 Å. Before data acquisition, all samples were placed at the same height, aligned with the top edge of the holder. GIWAXS analysis was conducted using aSAXS Focus 3.0 system equipped with an EIGER 2R 500 K detector at the incident angle of 0.3°. The x-ray radiation was generated from Cu X-ray Source (8.05 keV, 1.54 Å). The corresponding GIWAXS data reduction and visualization were made based on reported methods (*73*, *74*).

Steady-state (PL) and TRPL measurements were acquired using a time-correlated single-photon counting setup (FluoTime 300, PicoQuant). The samples were excited from the perovskite side using a 440-nm laser head (LDH-P-C-440, PicoQuant) and a 640-nm laser head (LDH-D-C-640, PicoQuant). The photoluminescence quantum yield (PLQY) was collected using an additional integrating sphere.

The charge carrier lifetime for perovskite under different modifications was identified by fitting the corresponding TRPL spectra using a stretched-exponential decay function (*36*)

$$I\left(t\right)=I_{0}e^{-{\left(t/\tau_{c}\right)}^{\beta }}$$ (2)

where *I*(*t*) represents the time-dependent PL intensity, *I*_0_ is the initial PL intensity, *t* is time, and τ_c_ denotes the characteristic lifetime taken after excitation for the PL intensity to drop to 1/*e* of its initial intensity (*I*_0_). β is the distribution coefficient.

The PL mapping of perovskite films was conducted using an IMATM Vis microscope (Photon etc.). The setup uses a Nikon 20×, 0.45 numerical aperture, chromatic aberration corrected objective collecting light onto a silicon complementary metal oxide semiconductor camera (Hamamatsu), allowing spatial resolution of light. Photoluminescence maps were performed using a 405-nm continuous-wave laser using a dichroic beam splitter to direct the laser onto the sample and remove the laser from the detected light.

The ultraviolet-visible (UV-vis) absorption spectroscopy spectra were collected using Varian Cary 5000 UV-vis-NIR spectrophotometer. The optical bandgap energy is derived from the *T*_auc_ plot analysis by extrapolating the linear fit of the onset of the absorption spectra. The exciton binding is obtained by running Elliott modeling of the onset of absorption spectra. In Eq. 3, the first term and second terms correspond to the 1-s excitonic transition states and unbounded continuum absorption state, respectively. The *E*_g_ is the bandgap energy, and *E*_x_ is the exciton binding energy. Band-to-band absorption occurs when a photon with energy equal to or greater than the bandgap energy of semiconductors excites an electron directly from the valence band to the conduction band. This process is intrinsic to semiconductors (*41*). Exciton absorption involves the formation of an exciton, which is a bound electron-hole pair resulting from Coulombic attraction. This process leads to the creation of discrete exciton states, which exist at energies slightly below the conduction band edge due to the exciton binding energy (*39*)

$$\begin{array}{c} \alpha \hbar \omega =A\cdot \theta \left(\hbar \omega -E_{g}\right)\cdot D_{\text{CV}}\cdot \left[\frac{\pi e^{\mathrm{\pi x}}}{\text{sin}h\left(\pi x\right)}\right]\\ +A\cdot E_{x}\sum_{n=1}^{\infty }\frac{4\pi }{{n_{\text{ex}}}^{3}}\cdot \delta \left(\hbar \omega -E_{g}+E_{x}/n^{2}\right) \end{array}$$ (3)

The electrostatic potentials (φ) were calculated using the Gaussian 09 package at B3lYP/6-31G(d) level. The maximum φ assigned to the NH_3_^+^ group was obtained by using a reported method (*75*).

The *J*-*V* measurements and MPP tracking were carried out using a Keysight 2400 Source Meter in conjunction with an AAA Enlitech SS-F5-3A solar simulator under AM 1.5G illumination. These measurements were performed outside of the glovebox under ambient laboratory conditions. Before each measurement, the solar simulator was calibrated using a standard monocrystalline silicon reference cell (Fraunhofer ISE CalLab, ISE001/013-2018) equipped with a KG-5 filter. The *J*-*V* measurements were conducted in both backward (1.2 to −0.2 V, step size: 0.02 V) and forward (−0.2 to 1.2 V, step size: 0.02 V) scanning directions, with no prelight soaking or prebiasing applied. Stabilized power output was determined at the MPP bias voltage for each device. Dark *J*-*V* measurements were performed under identical instrumental conditions, but with the light source deactivated. To define the active area of the solar cells, an aperture mask with an area of 0.09 cm^2^ was used. All devices were measured without encapsulation. The storage conditions for solar cells in stability tests are detailed in fig. S27. The EQE was measured in air by using an internal quantum efficiency system (Bentham PV300) under irradiation by a 100-W Xenon lamp ranging from 300 to 900 nm. All devices were measured without encapsulation.

The Mott-Schottky measurement was executed for solar cells in the dark by using Gamry Interface 1000 with a modulation frequency of 1 kHz and ac voltage of 10 mV, respectively. The built-in potential was extracted from Fig. 5D, based on Eq. 4. The *V*_R_ denotes the reverse bias, and *N*_a_ and *N*_d_ represent the accepter concentration and donor concentration, respectively

$$C^{-2}=\left\{\frac{2\left(V_{\text{bi}}+V_{R}\right)\left(N_{a}+N_{d}\right)}{q\varepsilon N_{a}N_{d}}\right\}$$ (4)

Contact angle measurement was conducted using a Drop Shape Analyzer (DSA25, KRŰSS GmbH). A 2-μl drop of DI water was placed on the perovskite surface and measured in air at room temperature.

## Supplementary Materials

### This PDF file includes:

Supplementary Notes S1 to S3 Figs. S1 to S28 Tables S1 to S4 References
