# Supplementary material for: Strain relaxation in halide perovskites via 2D/3D perovskite heterojunction formation
Source: Sci Adv. 2025 Jun 27;11(26):eadu3459. doi: 10.1126/sciadv.adu3459 (PMC12204152; doi:10.1126/sciadv.adu3459)
Supplement: Supplementary file 1 — Supplementary Notes S1 to S3 Figs. S1 to S28 Tables S1 to S4 References [file sciadv.adu3459_sm.pdf]

Science Advances
AAAS

Supplementary Materials for

Strain relaxation in halide perovskites via 2D/3D perovskite heterojunction formation

Dongtao Liu *et al.*

Corresponding author: Wei Zhang, wz0003@surrey.ac.uk; Lei Su, l.su@qmul.ac.uk

Sci. Adv. **11**, eadu3459 (2025)
DOI: 10.1126/sciadv.adu3459

This PDF file includes:

Supplementary Notes S1 to S3
Figs. S1 to S28
Tables S1 to S4
References

Supplementary Note S1

Strain heterogeneity along the perovskite thicknesses

To analyze strain heterogeneity along the perovskite thickness and its correlation with surface treatment, perovskite thin films of varying thicknesses were prepared on SnO_2/ITO substrates by controlling the precursor concentration (1.4 M, 0.70 M, and 0.35 M). The 1.4 M precursor, representing the primary setup in this work, produced films with a thickness of approximately 780 nm, as determined by cross-sectional SEM (Fig. 5A). These samples then underwent XRD characterization. Before strain analysis, all measured XRD patterns (Fig. S11A) were corrected using the ITO substrate diffraction profile. XRD analysis (Fig. S11, B and C) revealed that thinner films (denoted as 0.35 M) exhibit a negative shift in the Bragg peak compared to thicker films (denoted as 0.7 M and 1.4 M), indicating reduced tensile strain in thinner films. This trend implies that for our primary perovskite setup, the perovskite at the bottom interface (represented by 0.35M perovskite) experiences less tensile strain than the bulk crystal (represented by 0.7M and 1.4M perovskite). Optical analysis further consolidates this, showing a bandgap reduction of 31meV for 0.35M perovskite films compared to 1.4M perovskite films (Fig. S11D and Fig. S12), consistent with their lower tensile strain.

Strain heterogeneity along perovskite film thickness has been investigated, yet there is no consensus on whether tensile strain predominantly accumulates at the bottom interface or top surface(*12, 14*). Considering that the discrepancies between these reports may stem from variations in perovskite crystallization processes, SEM analysis was conducted on our thin (0.35 M) and thick (1.4 M) perovskite films. In Fig. S11, E and F, thicker films exhibit a compact, uniform morphology, whereas thinner films show a discontinuous grain distribution with voids and exposed SnO_2/ITO areas, consistent with prior studies(*76*). As discussed, tensile strain primarily originates from the thermal expansion mismatch between the perovskite layer and the substrate. However, insufficient inter-grain bonding and non-compact perovskite film morphology can negate substrate-induced lattice constraints(*29*), resulting in reduced tensile strain and thus a negative Bragg peak shift in halide perovskites. Consequently, in line with our observation, it is hypothesized that increasing film thickness or transitioning the bottom interface to the top surface could enhance tensile strain accumulation by improving film compactness and crystal integrity(*77, 78*).

After ligand treatment, notable tensile strain relaxation (characterized by the peak shifts between modified perovskite and control perovskite) and bandgap redshifts were observed in thicker films (0.7M and 1.4M), whereas thinner films (0.35M) showed negligible changes in strain and bandgap (Fig. S11D and Fig. S12). This highlights that thinner films (or at the bottom interface in the case of our primary perovskites) are inherently less tensile-strained, and their strain levels are less responsive to surface treatment. Surface treatment-induced strain relaxation is effective only when tensile strain is already present in perovskite films (or at the top surface in the case of our primary perovskites) with a higher film compactness and crystal integrity.

Supplementary Note S2

Theoretical calculation of strain-induced Bragg peak shift

Driven by the Poisson effect(*42*), the development of in-plane tensile strain (stress) leads to an increase in compressive strain in the out-of-plane direction(*16*). Considering elastic deformation, the residual tensile stress resulting from thermal expansion mismatch can be determined using equation S1(*31, 77, 78*).

$$\sigma = \frac{E\Delta\alpha\Delta T}{1-\nu} \qquad \text{(S1)}$$

Here, $\Delta\alpha$ represents the difference in the coefficient of thermal expansion between glass (10×10^{-6} °C^{-1}) and perovskites (50×10^{-6} °C^{-1})(*31*), while ΔT denotes the temperature difference between annealing temperature (100°C) and room temperature (25°C). Young's modulus (E) and Poisson ratio (ν) for perovskites are ~10-15 Gpa(*31*) and 0.33(*77*), respectively.

Given the presence of equi-biaxial in-plane stress in thin-film perovskites, the relationship between in-plane strain (ε_x) and stress (σ_x) is described as follows. The in-plane strain is determined by substituting the stress calculated from equation S1 into equation S2(*77*).

$$\frac{\sigma_x}{\varepsilon_x} = \frac{E}{1-\nu} \qquad \text{(S2)}$$

Equation S3 defines the relationship between in-plane strain and out-of-plane strain. Consequently, the out-of-plane strain (ε_z) can be determined using equation S3, with in-plane strain (ε_x) obtained from equation S2(*79*). Last, the theoretical Bragg peak shifts ($\Delta\theta$) can be ascertained based on equation S4(*55*), with ε_z obtained from the equation S3. The visualization of the calculation result was given in Fig. S13.

$$\varepsilon_z = \frac{2\nu}{1-\nu}\varepsilon_x \qquad \text{(S3)}$$

$$\varepsilon_z = -\Delta\theta\cot\theta \qquad \text{(S4)}$$

Supplementary Note S3

Phase transition induced strain vibration

In contrast to our expectation, (001) peak in OA(PA)-60H is surprisingly positively shifted by ~0.003°-0.014° compared to OA(PA)-60L (Fig. S14 and Table S1), respectively, which implies a rather slightly increased tensile strain in OA(PA)-60H. Previous research indicated that dissimilar to tensile strain induced by interfacial thermal expansion mismatch, tensile strain can also be promoted in halide perovskite, such as $CsPbI_{2.7}Br_{0.3}$, through α-to-$\beta(\gamma)$ phase transition and anisotropic texture propagation(*11*). At room temperature, the reported $CsPbI_{2.7}Br_{0.3}$ remains in black phase similar to our perovskites. On the other hand, cell volume changes during phase transitions can contribute to tensile strain, for instance, halide perovskites expand during the α-to-β phase transition(*80*). In our XRD patterns, (001) peak splitting was observed in both PA-60H and OA-60H (Fig. S14), accompanied by the detection of a faint (110) shoulder peak(*56, 81*). A more obvious peak splitting was also determined in (002) plane. These peak splitting have been regarded as a result of α-to-β phase transition(*13, 56, 81*). Accordingly, the unexpected strain variation observed in OA(PA)-60H is likely due to the partial phase transition.

Besides, upon the α-to-β phase transition in OA(PA)-60H, their optical properties would be rather reduced because the tetragonal domain in halide perovskites is reported to have a reduced absorption coefficient compared to the cubic domain(*56*). This suggests that radical tensile strain relaxation involved with phase transition could also be detrimental for perovskites in optoelectronic applications.

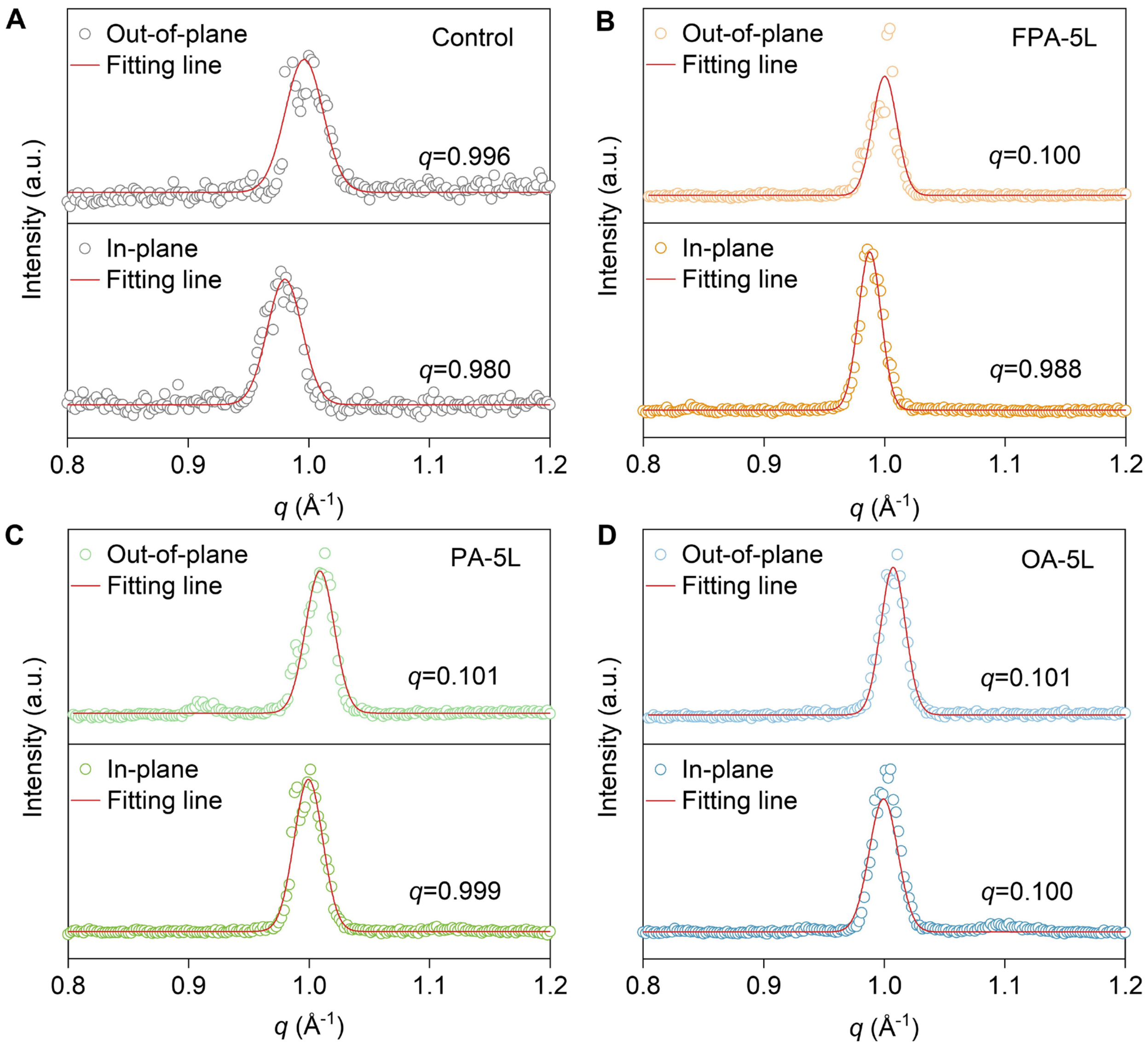

Fig. S1. 1D GIWAXS profile analysis for perovskites. 1D GIWAXS for the (**A**) control, (**B**) FPA-5L, (**C**) PA-5L and (**D**) OA-5L, respectively. The 1D profile is extracted based on the sector integrity of Fig. 1. The q vector is identified based on Gaussian fitting. The cake integrity along the q_z and q_{xy} are associated with the (001) plane of out-of-plane and in-plane direction, respectively. The expansion of in-plane lattice ($q_{xy} < q_z, q = 2\pi/d$) indicates the generation of in-plane tensile strain in halide perovskites.

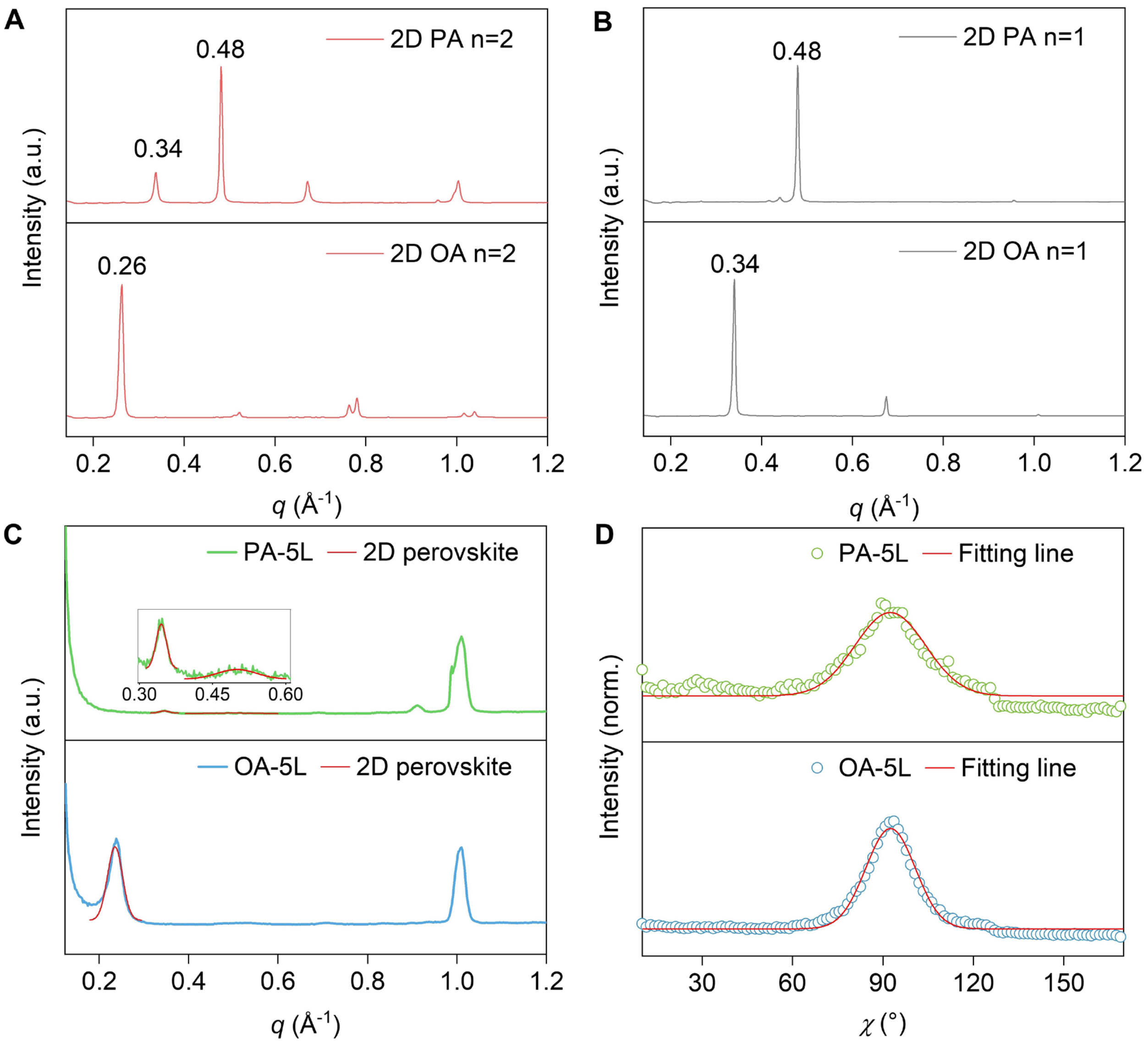

Fig. S2. Crystallographic analysis for 2D perovskites. (**A**) XRD profile for $(PA)_2(FA)Pb_2X_7$ and $(OA)_2(FA)Pb_2X_7$, n=2, respectively. (**B**) XRD profile for $(PA)_2PbX_4$ and $(OA)_2PbX_4$, n=1, respectively. (**C**) The 1D profile derived from sector integral of χ=10-90° for 2D GIWAXS. The q vector for 2D perovskites is identified based on Gaussian fitting. The formation of the 2D perovskite phase within the 2D/3D perovskite, including OA(PA)-5L, closely aligns with the XRD profiles characteristic of pure 2D perovskites with n=2. (**D**) Azimuthal angle scans of the corresponding 2D perovskite crystal plane in GIWAXS.

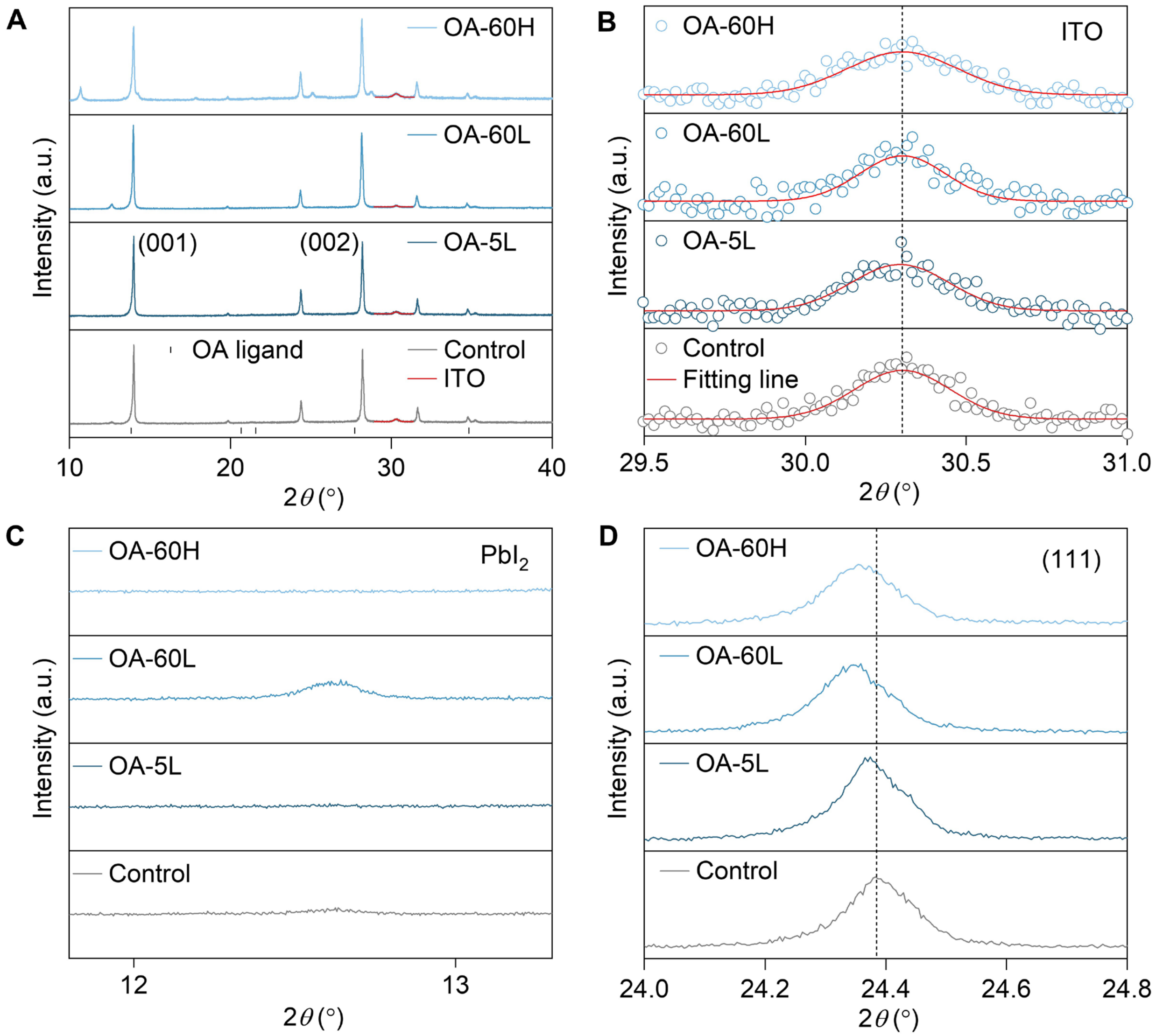

Fig. S3. XRD measurement for OA-modified perovskites. (**A**) XRD patterns for control perovskite and OA-modified 3D perovskites under different conditions. The dashed line in the XRD profile represents pure OA ligand extracted from Ref.(*82*). (**B**) ITO correction and (**C**) PbI_2 XRD profile for control perovskite and OA-modified 3D perovskites under different conditions. The ITO peak position is identified based on Gaussian fitting. (**D**) XRD profile of (111) plane for control perovskite and OA-modified 3D perovskites under different conditions. Pronounced negative shifts in the (111) peak was observed in OA-modified samples, for example, $\Delta 2\theta$ reaches 0.014° between OA-5L and control.

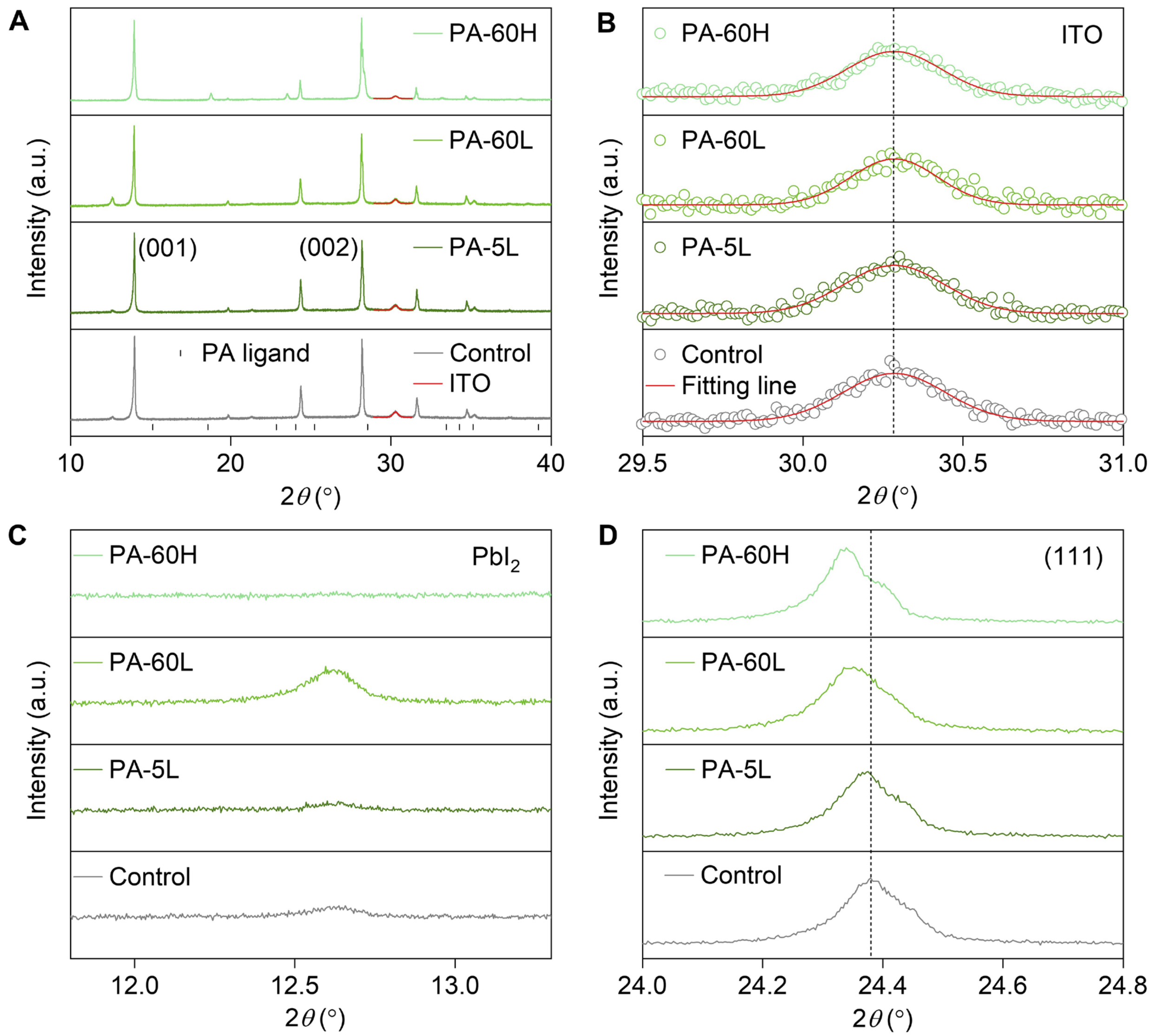

Fig. S4. XRD measurement for PA-modified perovskites. (**A**) XRD patterns for control perovskite and PA-modified 3D perovskites under different conditions. The dashed line in the XRD profile represents pure PA ligand extracted from Ref.(*83*) (JCPDS No. 00-040-1924). (**B**) ITO correction and (**C**) PbI_2 XRD profile for control perovskite and PA-modified 3D perovskites under different conditions. The ITO peak position is identified based on Gaussian fitting. (**D**) XRD profile of (111) plane for control perovskite and PA-modified 3D perovskites under different conditions. Pronounced negative shifts in the (111) peak was observed in PA-modified samples, for example, $\Delta 2\theta$ reaches 0.011° between PA-5L and control.

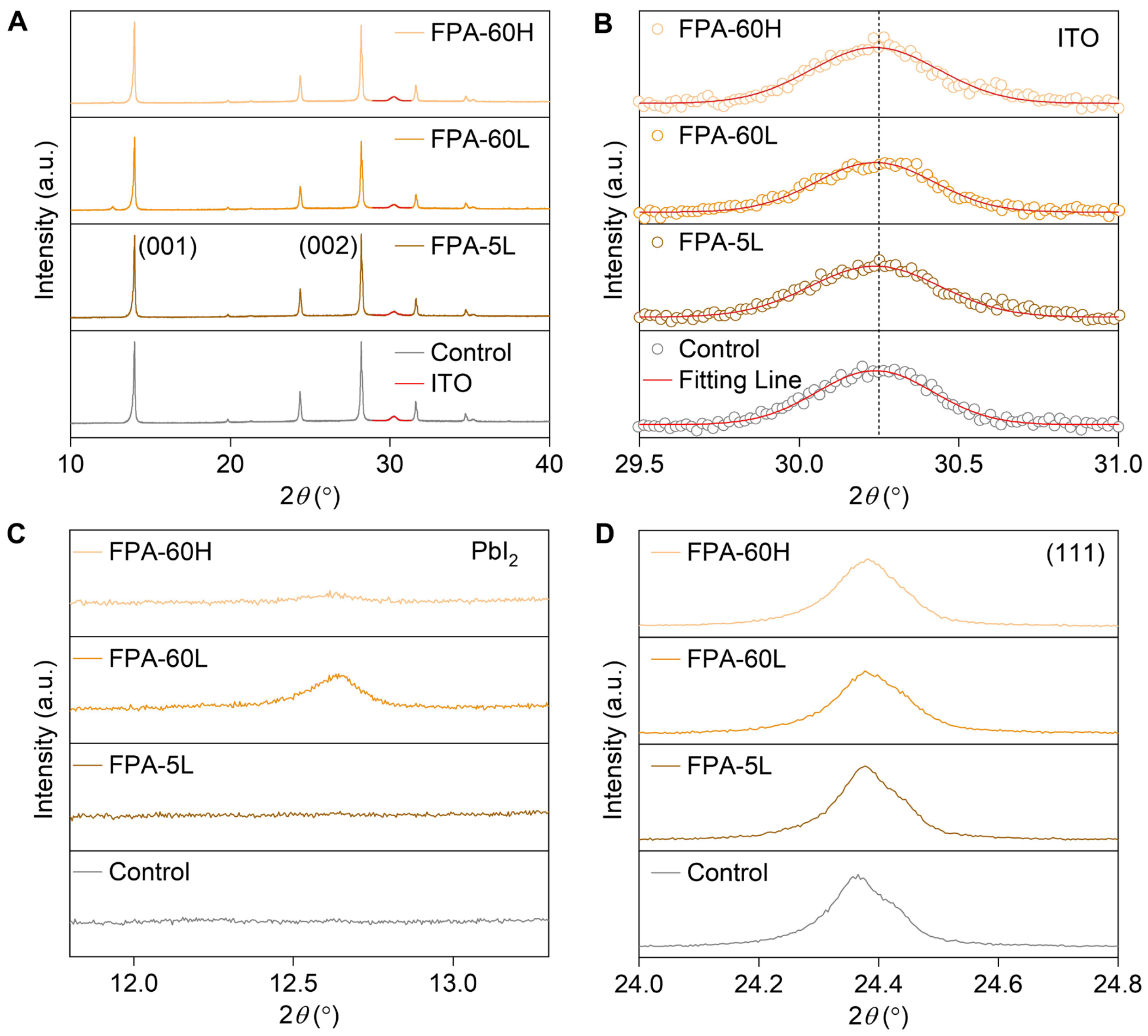

Fig. S5. XRD measurement for FPA-modified perovskites. (**A**) XRD patterns for control perovskite and FPA-modified 3D perovskites under different conditions. (**B**) ITO correction and (**C**) PbI_2 XRD profile for control perovskite and FPA-modified 3D perovskites under different conditions. The ITO peak position is identified based on Gaussian fitting. Notably, it is found that the PbI_2 peak intensity in XRD is slightly increased in FPA-60L (Fig. S5C) while there is no evidence that tensile strain relaxation is improved in FPA-60L. Such observation confirms that the slight generation of PbI_2 cannot cause tensile strain to be relaxed in perovskites. (**D**) XRD profile of (111) plane for control perovskite and FPA-modified 3D perovskites under different conditions.

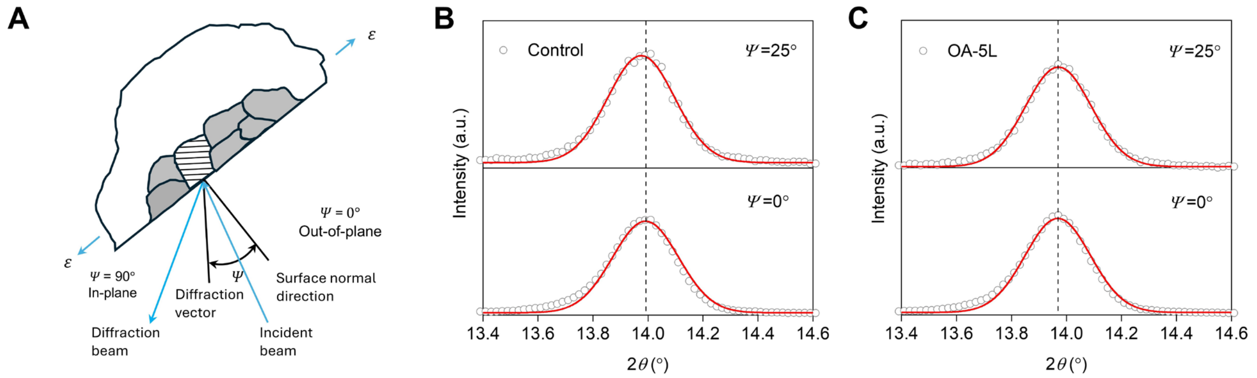

Fig. S6. GIXRD measurement for OA-modified perovskite and control perovskite. (**A**) Schematic illustration of GIXRD sampling geometry used for tensile strain evaluation (*42*). XRD profiles of the control (**B**) and OA-modified (**C**) perovskites obtained at different Ψ angles. For the control perovskite, the (001) diffraction peak shifts towards lower diffraction angles with increasing Ψ, indicating tensile strain in the in-plane lattice relative to the out-of-plane orientation. In contrast, the OA-modified perovskite exhibits negligible shifts in the (001) peak position between Ψ =0° and 25°, suggesting relaxed biaxial lattice deformation and effective in-plane tensile strain relaxation.

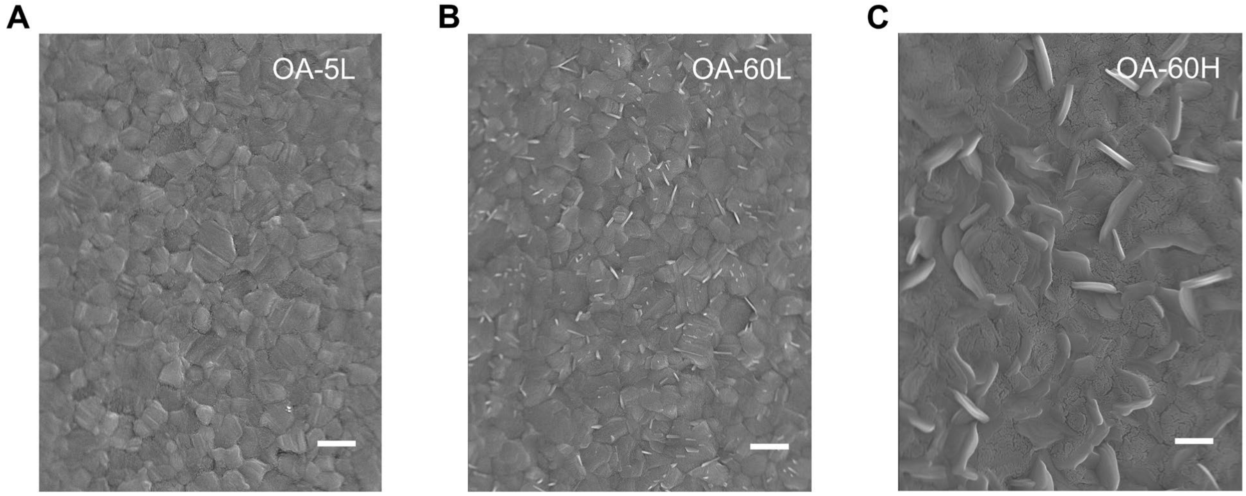

Fig. S7. SEM images for OA-modified perovskites. Scale bar: 1μm.

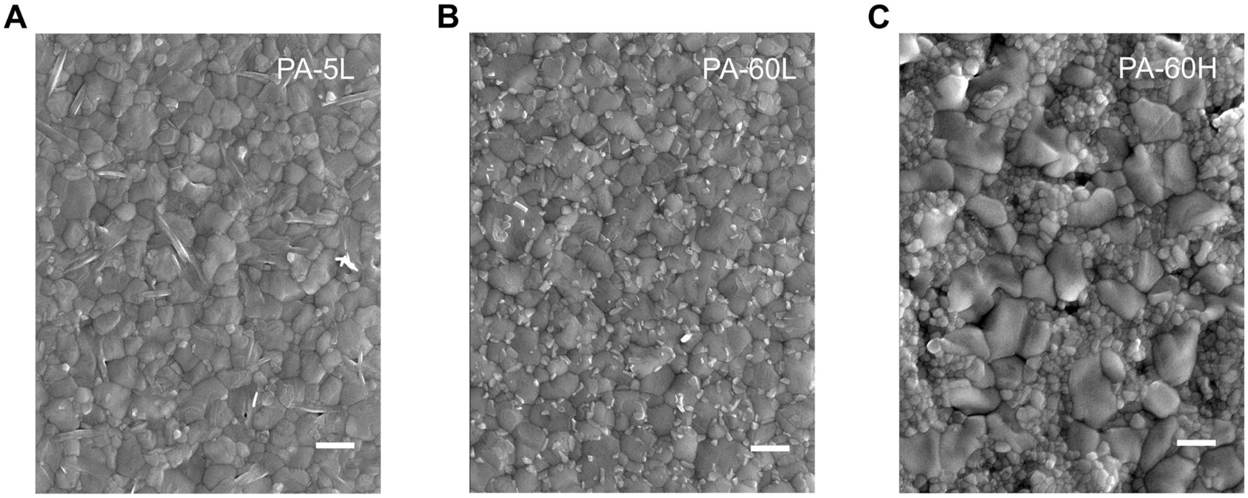

Fig. S8. SEM images for PA-modified perovskites. Scale bar: 1μm.

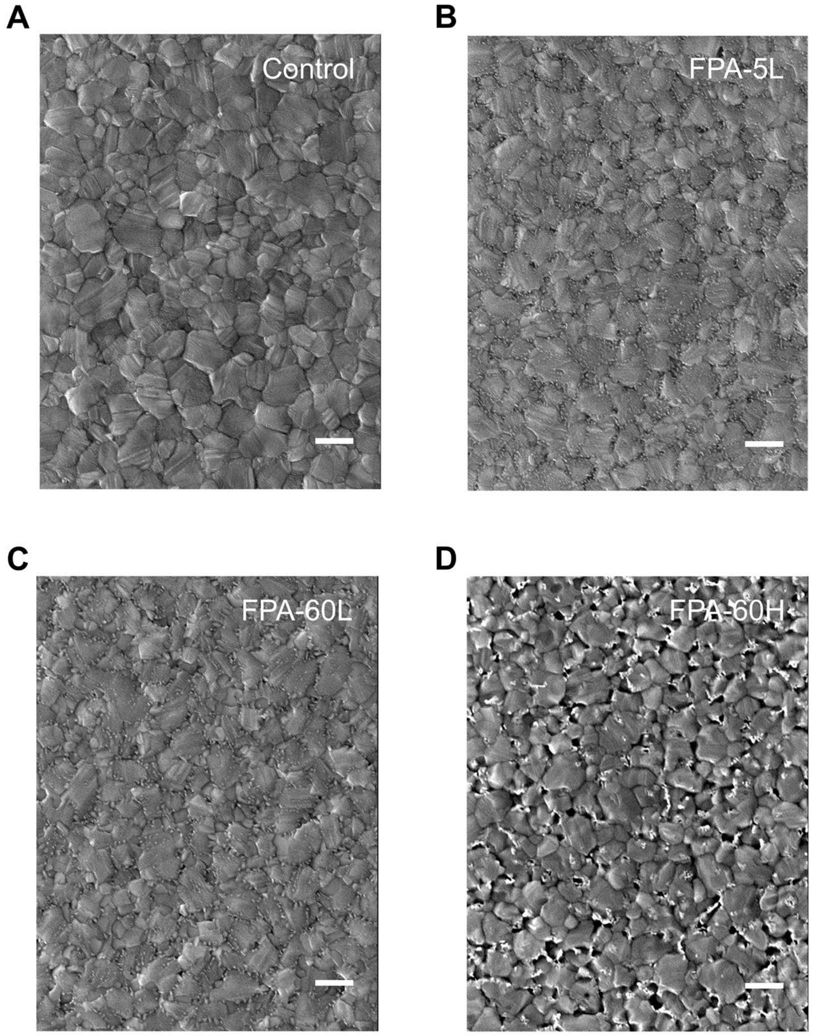

Fig. S9. SEM images for control perovskite and FPA-modified perovskites. Scale bar: 1μm.

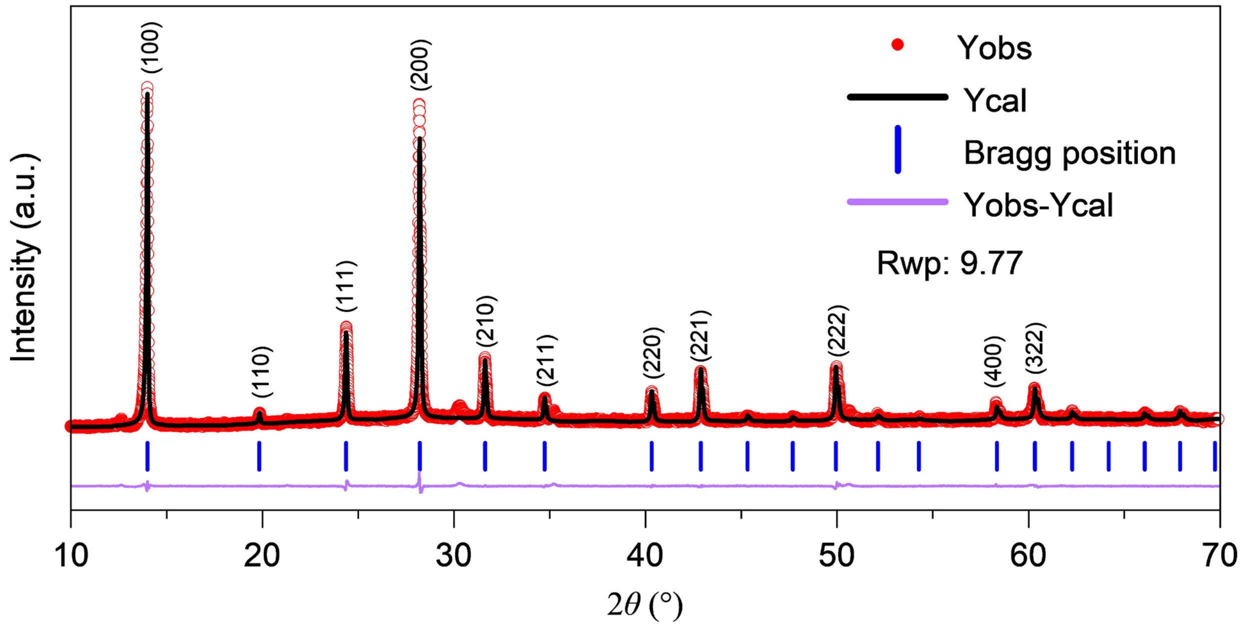

Fig. S10. XRD Rietveld refinement for control perovskite. The perovskite has a cubic phase structure, with a lattice parameter of a=b=c= 6.319Å, $\alpha=\beta=\gamma=90°$. By comparing the XRD patterns between control perovskites and modified perovskites (Fig. S3 to S5), it can be confirmed that in addition to the peaking splitting found in OA(PA)-60H, the XRD patterns across the samples predominantly exhibit characteristics consistent with a cubic phase structure.

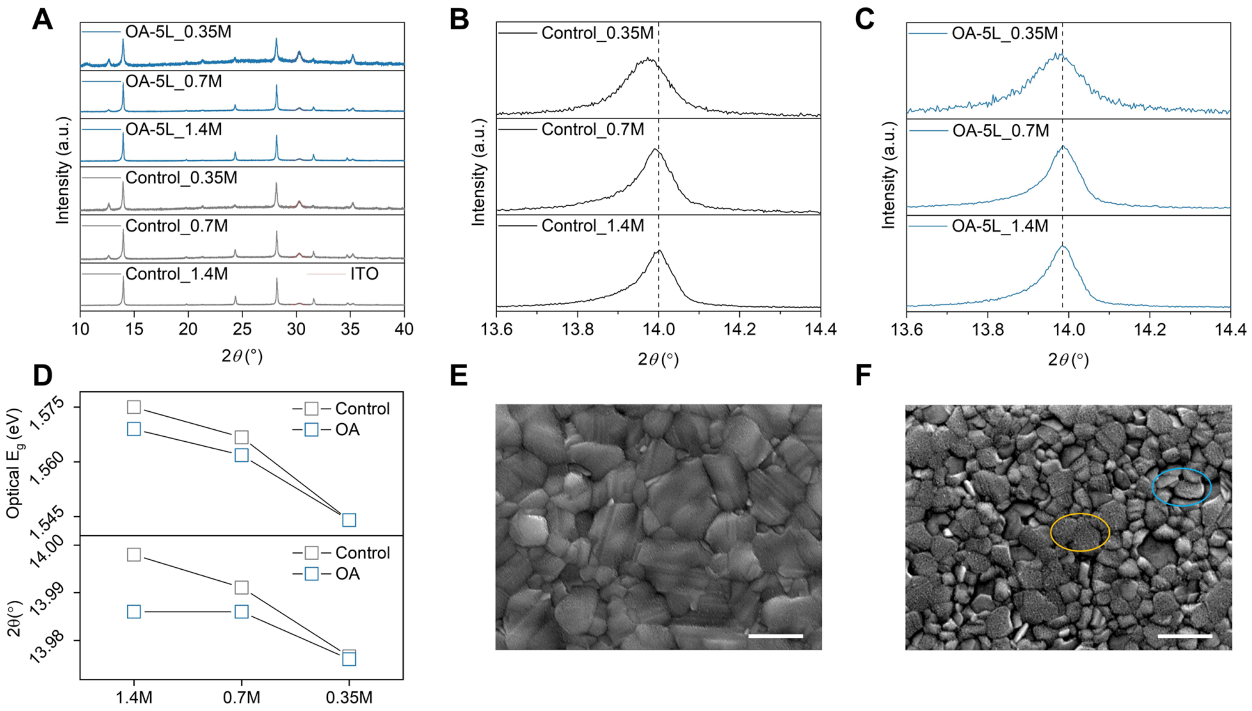

Fig. S11. Strain analysis for perovskites of different thicknesses. (**A**) XRD patterns for control and OA-modified perovskites under different conditions, where the 3D perovskite is prepared using precursors of different concentrations. The (001) plane XRD profile for control perovskites (**B**) and OA-modified 3D perovskites (**C**) under different conditions. (**D**) Summary of Bragg peak position and bandgap energy for control and OA-modified perovskites under different conditions. The bandgap is derived from the T_{auc} plot analysis of the absorption spectra, detailed in Fig. S12. SEM image of 1.4M perovskite film (**E**) and 0.35M perovskite film (**F**). Scale bar: 1μm. The yellow and blue circles delineate the perovskite and the exposed substrate regions, respectively.

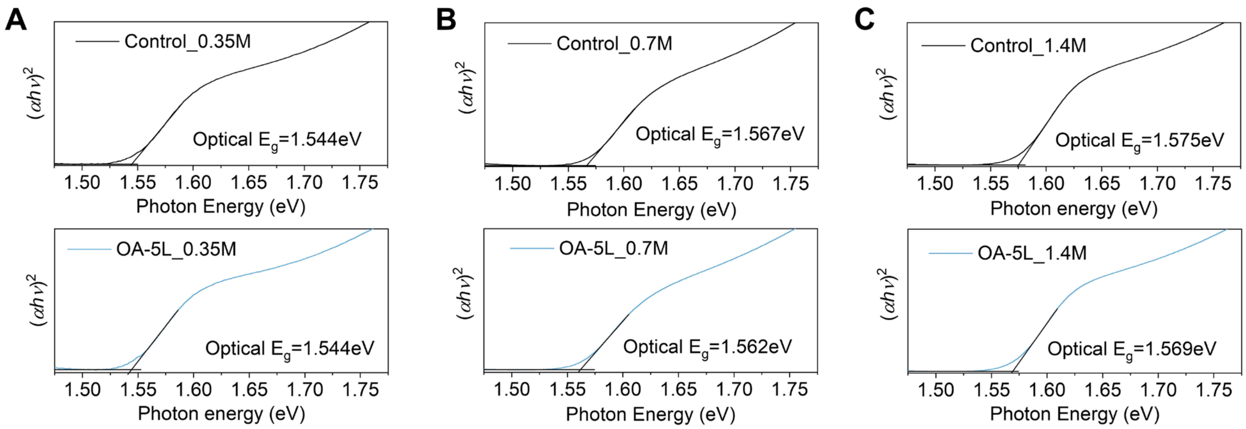

Fig. S12. UV-vis absorption spectra for perovskites of different thicknesses. The T_{auc} plot method is adopted to determine the bandgap energy.

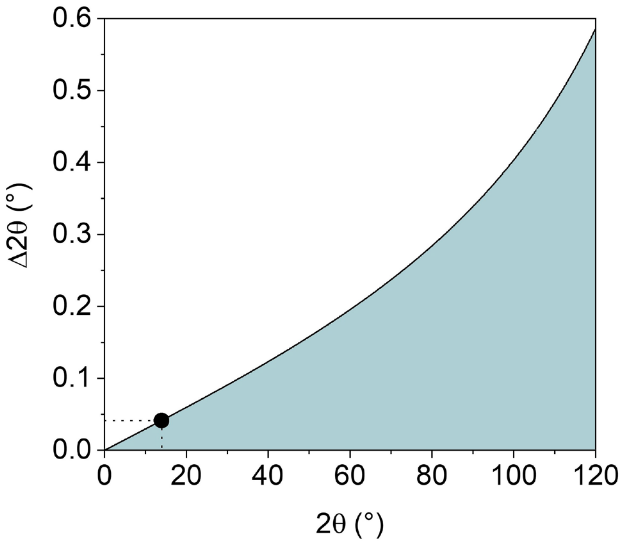

Fig. S13. Bragg peak shift for perovskites under calculated strain. The calculation process is detailed in Supplementary Note S2. Fig. 2B presents an alternative representation of the data shown in Fig. S13, focusing on the range of 2θ=0°-28°.

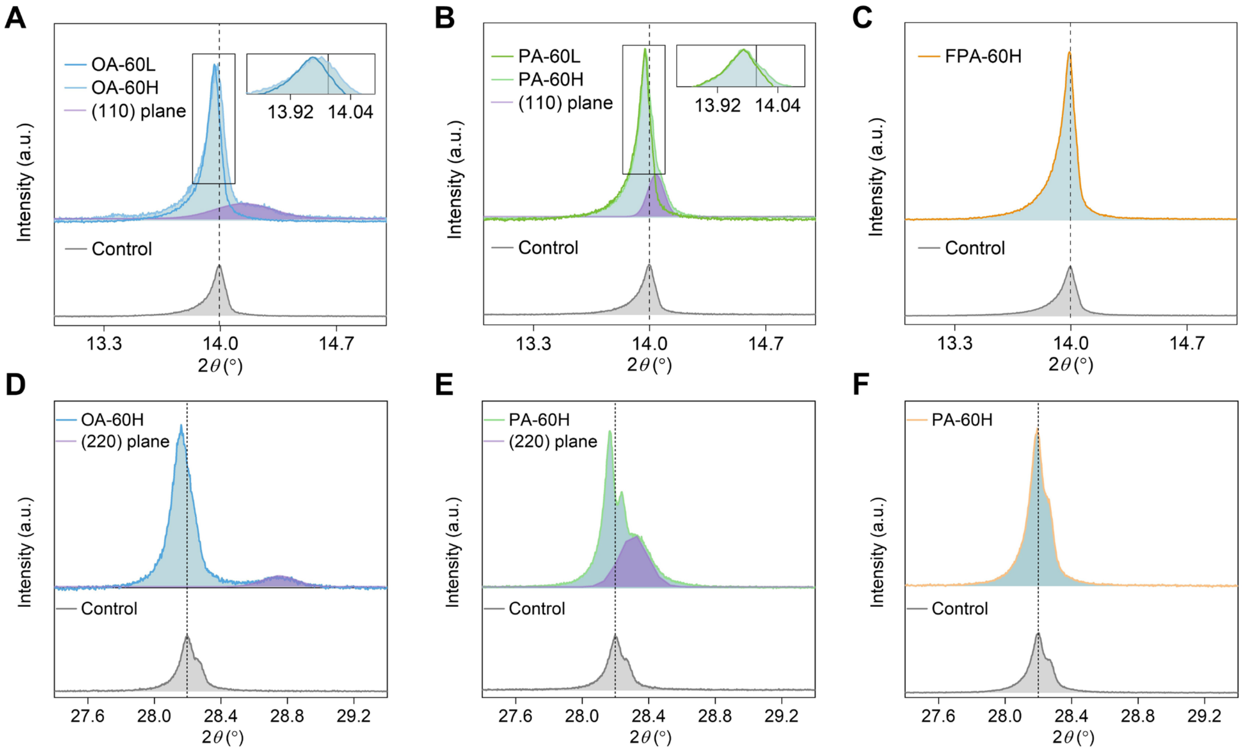

Fig. S14. Phase transition perturbed strain variation in halide perovskites. XRD profile of (001) plane in (**A**) OA-60H, (**B**) PA-60H and (**C**) FPA-60H. XRD profile of (002) plane in (**D**) OA-60H, (**E**) PA-60H and (**F**) FPA-60H. The inset enlarged XRD patterns are to highlight the peak shifts. The (110) and (220) peak is dyed purple.

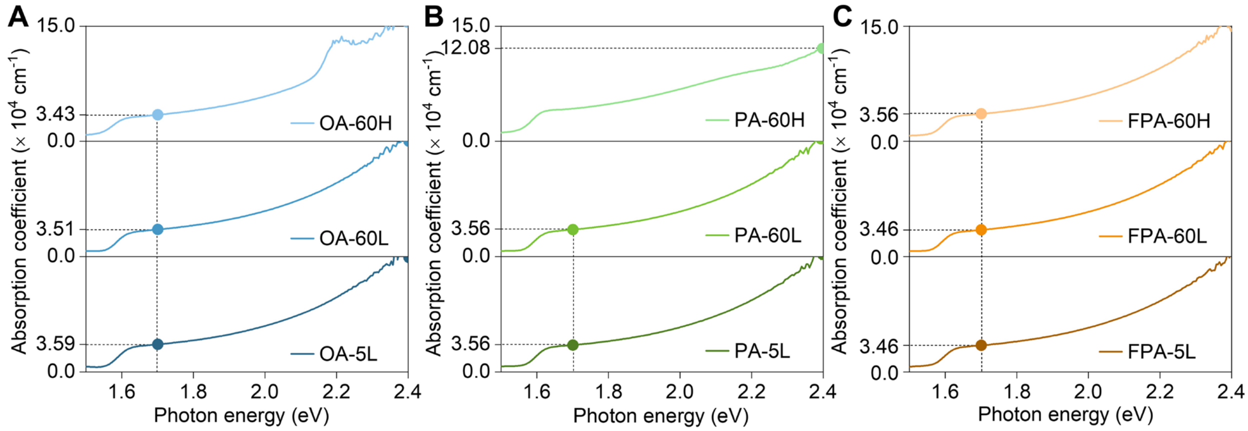

Fig. S15. UV-vis spectra for control and modified perovskites. The absorption coefficients at 1.7eV and 2.4eV were marked in the spectra. OA-60L(60H) presents a decreased absorption coefficient at 1.7eV compared to OA-5L. The absorption coefficient at 2.4eV remains nearly unchanged among OA-modified samples. For PA-modified samples, PA-60H has a notable drop in the absorption coefficient at 2.4eV. The absorption coefficient between 1.7eV and 2.4eV shows marginal variation across FPA-modified samples.

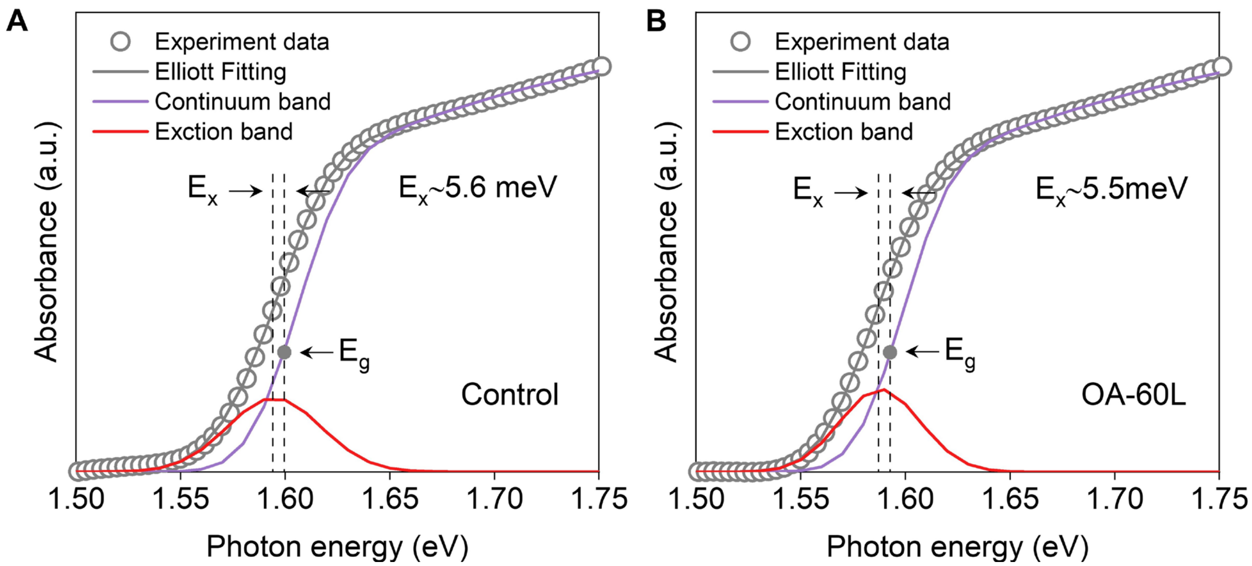

Fig. S16. Elliot's fitting to absorption spectra for the control and OA-modified perovskites.

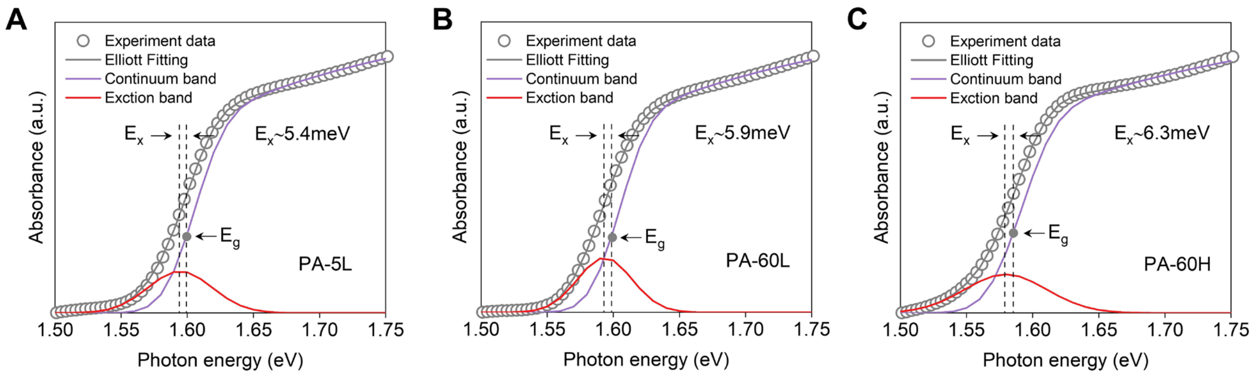

Fig. S17. Elliot's fitting to absorption spectra for PA-modified perovskites.

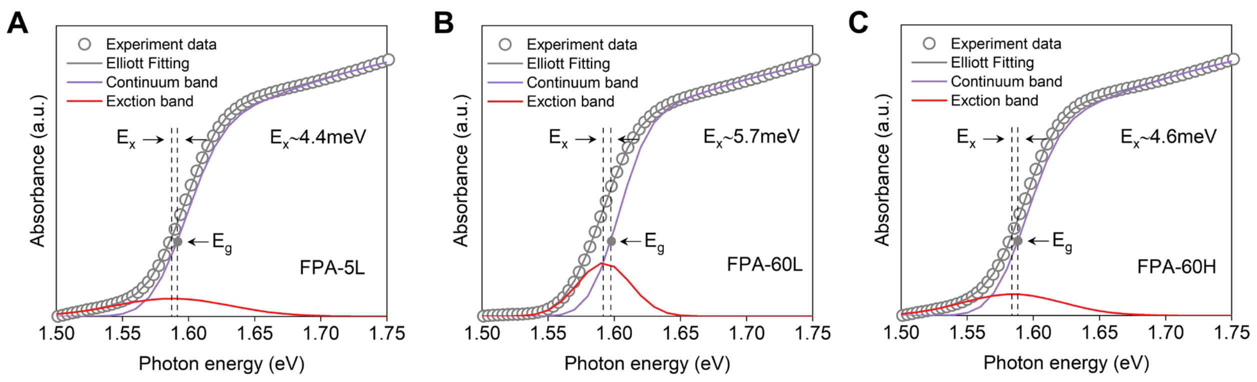

Fig. S18. Elliot's fitting to absorption spectra for FPA-modified perovskites.

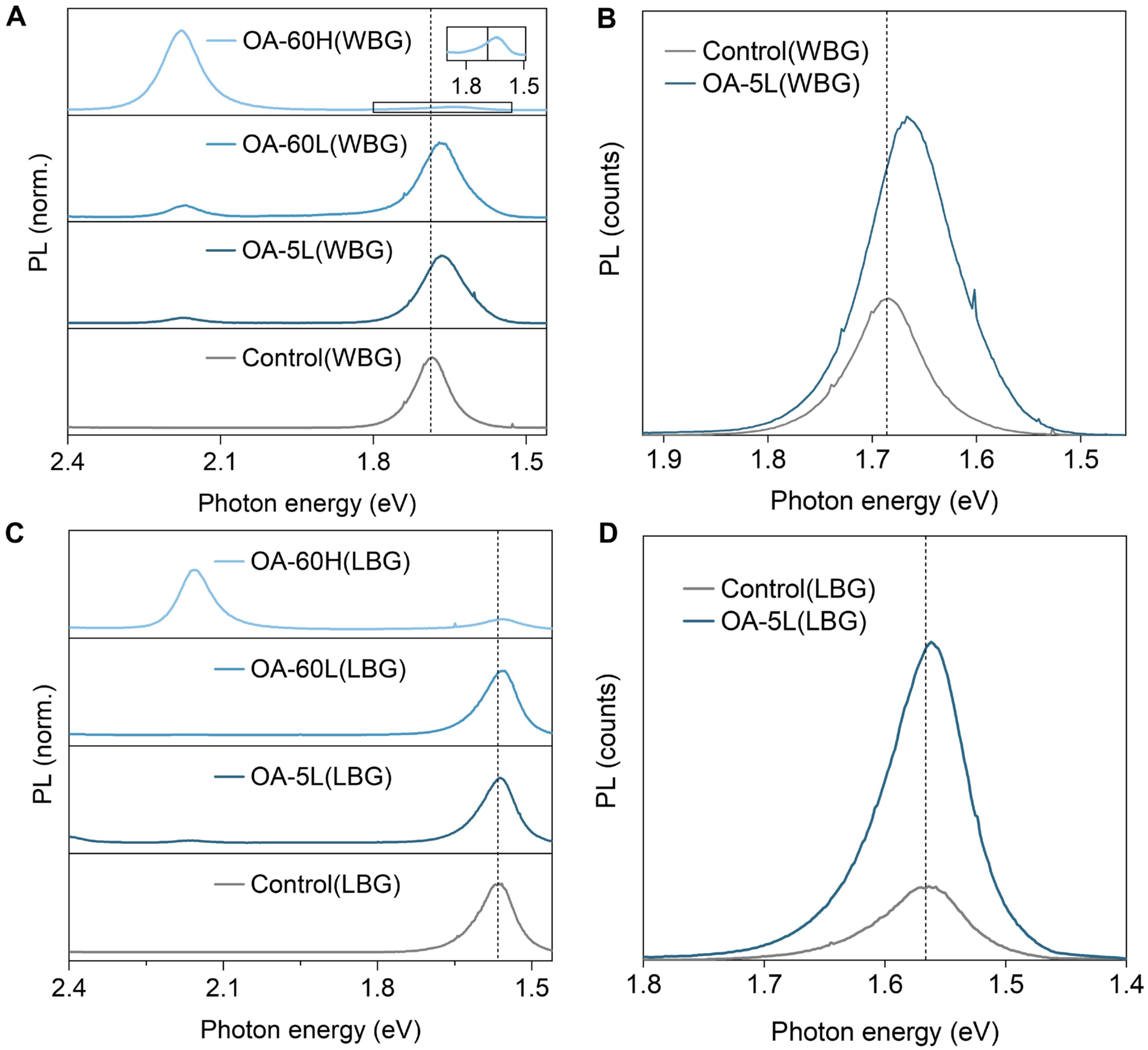

Fig. S19. Optical properties for OA-modified wide and low bandgap perovskites. (**A**) Normalized PL spectra for control and OA-modified wide bandgap (WBG) perovskites. (**B**) PL spectra for control and OA-5L(WBG). (**C**) Normalized PL spectra for control and OA-modified low bandgap (LBG) perovskites. (**D**) PL spectra for control and OA-5L(LBG). Notable PL redshifts are observed in OA-modified perovskites compared to control samples. Besides, the OA-5L preserves the most notable promotion in their PL intensity against the control samples, which is consistent with PL analysis in the main context where the moderate tensile strain suppresses the nonradiative recombination and enables broadened bandgap absorption for perovskites.

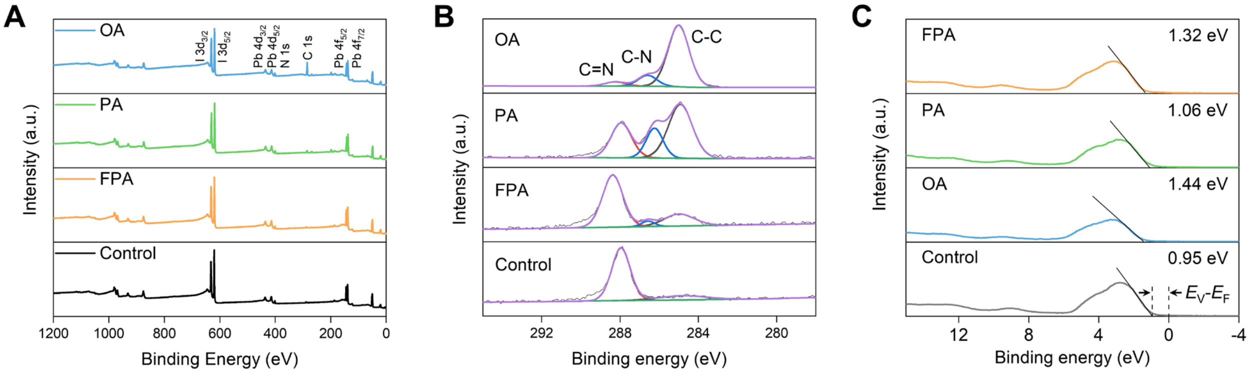

Fig. S20. XPS analysis for control and modified perovskites. (**A**) XPS survey spectra. (**B**) C1s XPS spectra. The increase of C-C peak intensity in modified perovskites indicates the successful coating of the ligand onto the perovskite surface. (**C**) Valence electron XPS spectra for control and modified perovskites. The linear fitting of the onset of spectra is to extract the energy offset between the Fermi level and the valence band maximum of perovskites.

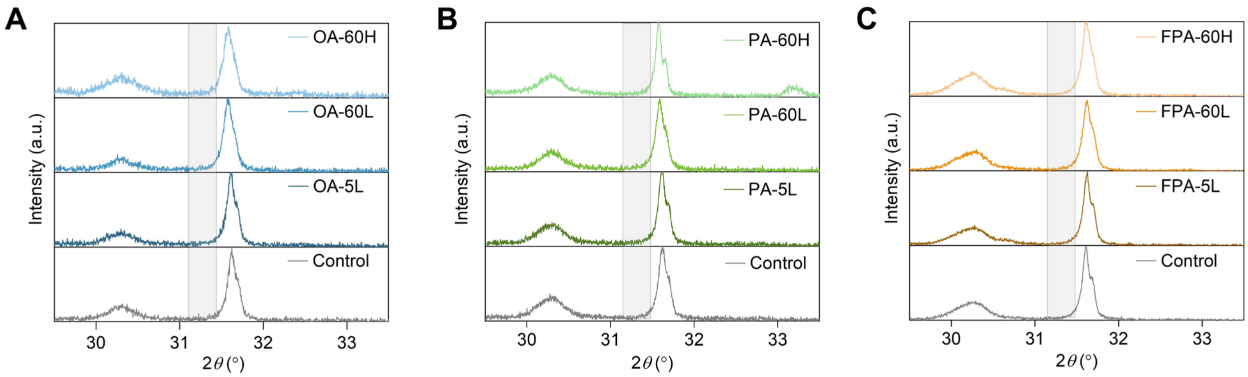

Fig. S21. Potential Pb^0 formations in XRD analysis for control and modified perovskites. It is noticed that there is no additional peak raised in the dyed region *(46)*, suggesting the appearance of Pb^0 is substantially low and limited to trace level.

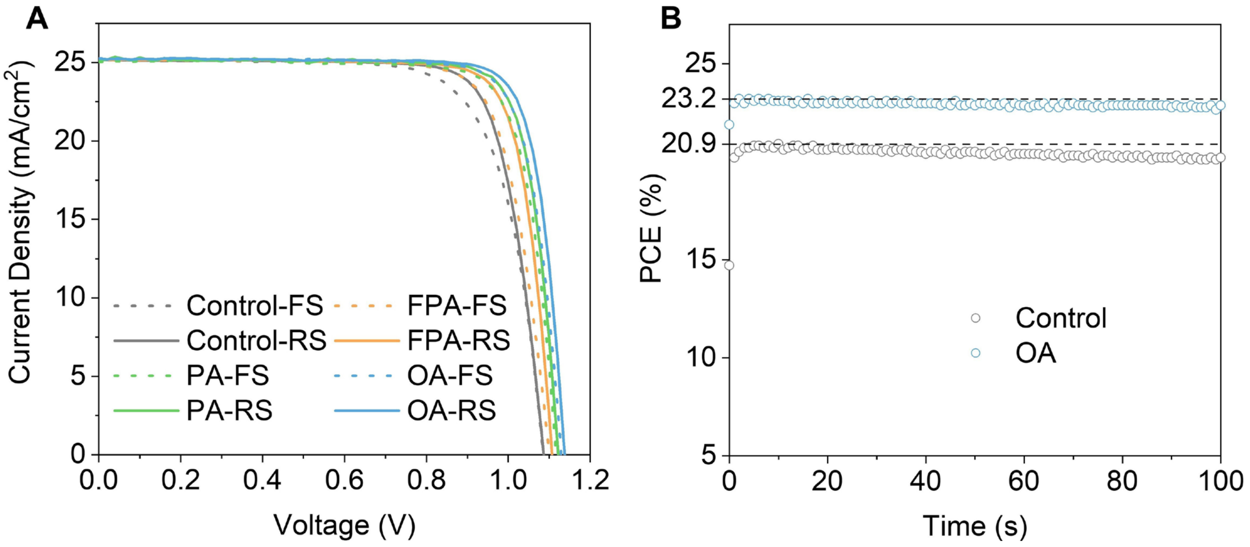

Fig. S22. Photovoltaic characterization for control and modified solar cells. (**A**) J−V curves by reverse (RS) and forward scan (FS) for best-performing perovskite solar cells in each group. (**B**) MPP tracking under 1 sun in air for control and OA-modified perovskite solar cells.

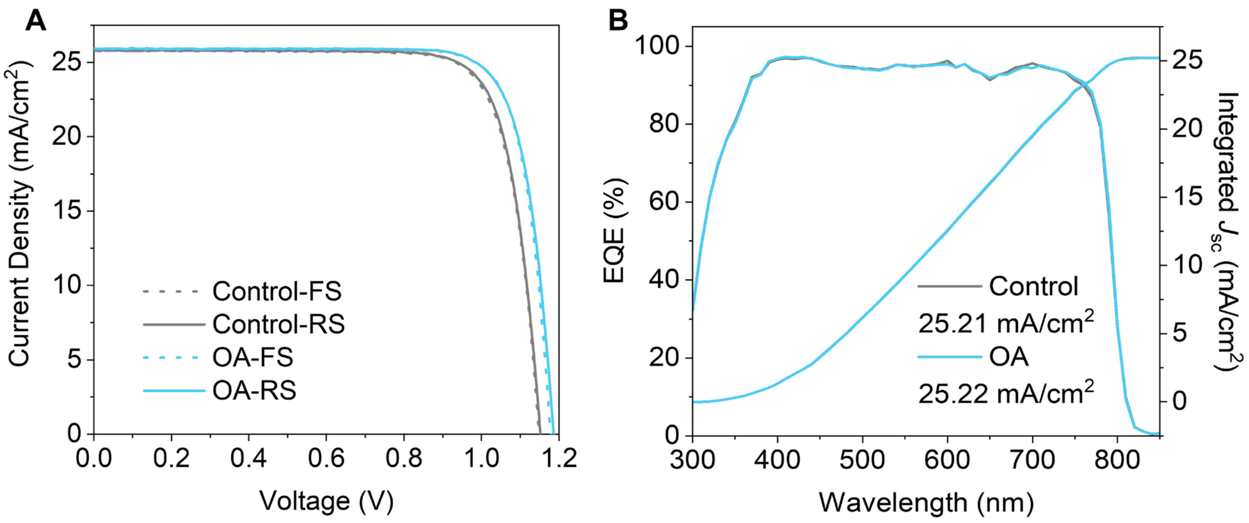

Fig. S23. Photovoltaic characterization for control and modified solar cells based on low bandgap perovskites. (**A**) J−V curves by reverse (RS) and forward scan (FS) for control and OA-modified perovskite solar cells. (**B**) EQE measurement in air for control and OA-modified perovskite solar cells.

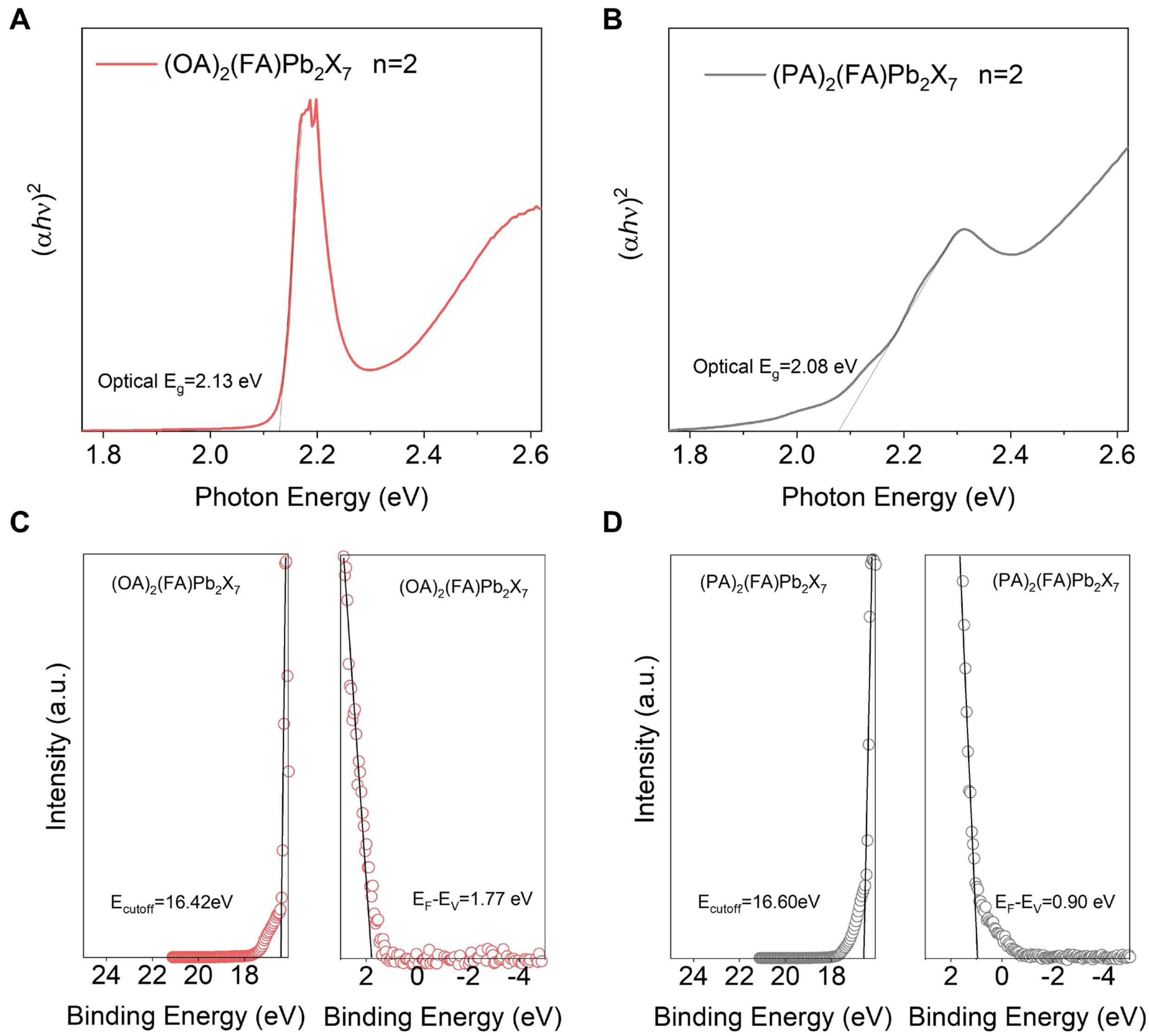

Fig. S24. Band structure analysis for 2D perovskites. Absorption spectra for **(A)** $(OA)_2(FA)Pb_2X_7$ and **(B)** $(PA)_2(FA)Pb_2X_7$, respectively. UPS spectra for **(C)** $(OA)_2(FA)Pb_2X_7$ and **(D)** $(PA)_2(FA)Pb_2X_7$, respectively.

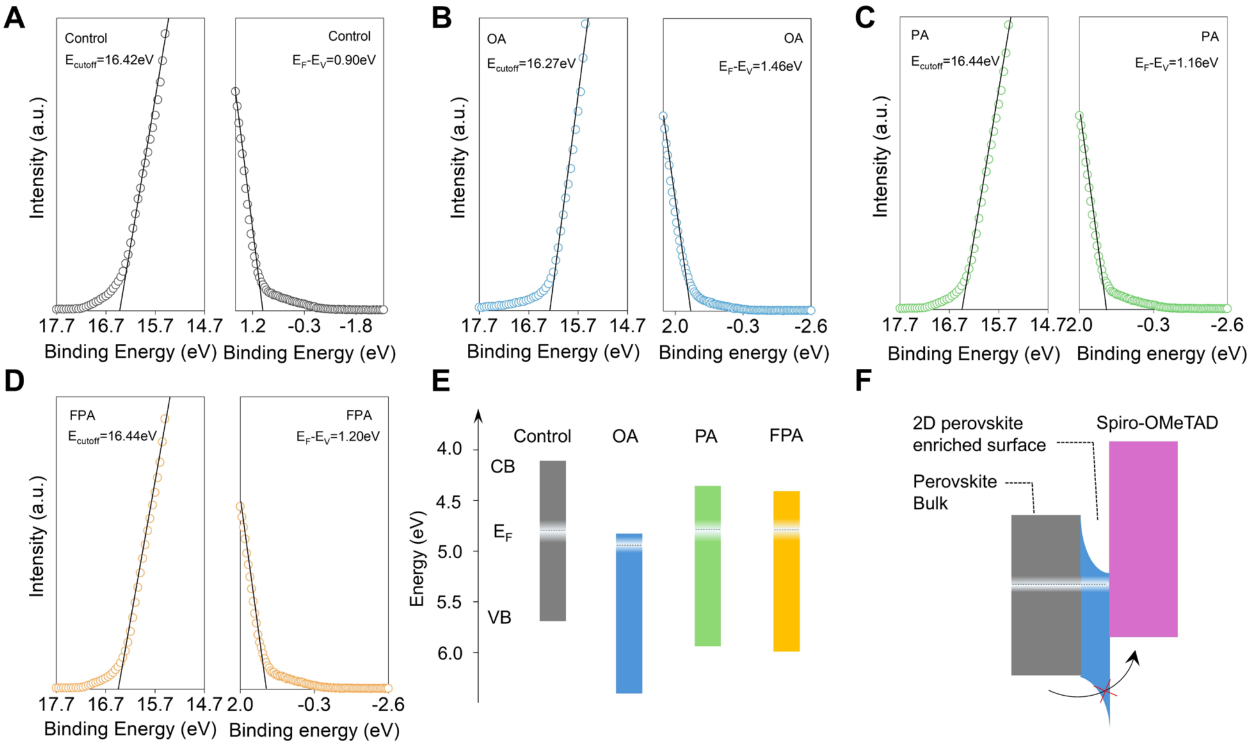

Fig. S25. Band structure analysis for control and modified 3D perovskites. UPS spectra for **(A)** control, **(B)** OA-5L, **(C)** PA-5L and **(D)** FPA-5L, respectively. **(E)** UPS revealed band structure for control and modified 3D perovskites. **(F)** Potential band bending following surface treatment. Here the 3D perovskite is set as the main entity for analysis, with the ligand acting as an electronic modifier. Ligand treatment induces an upward shift in the Fermi level of the 3D perovskite. As the ligand is applied via spin-coating, it, along with any resulting 2D perovskite phases, should predominantly accumulate at the surface, with certain diffusion into the bulk. Consequently, the strongest electronic modification and thus the largest Fermi level shift should occur at the surface, gradually diminishing with depth. These surface-localized modifications may result in a downward shift of the conduction and valence bands relative to the bulk.

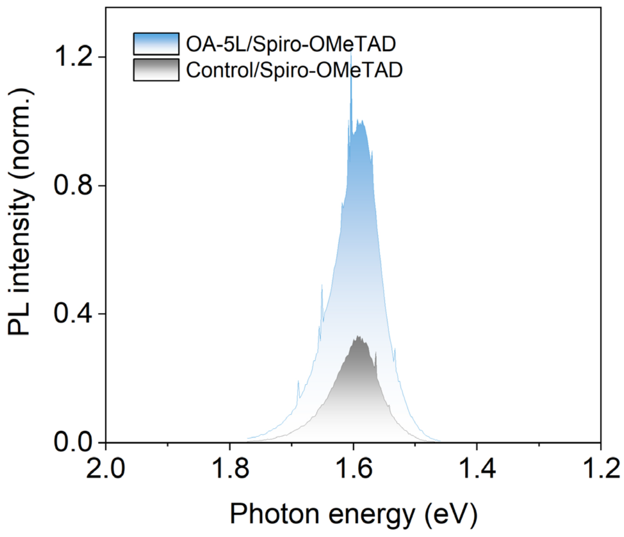

Fig. S26. PL measurement for half-stack device of control/Spiro-OMeTAD and OA-5L/ Spiro-OMeTAD. The 640 nm laser beam is adjusted to one-sun equivalent illumination as the excitation source.

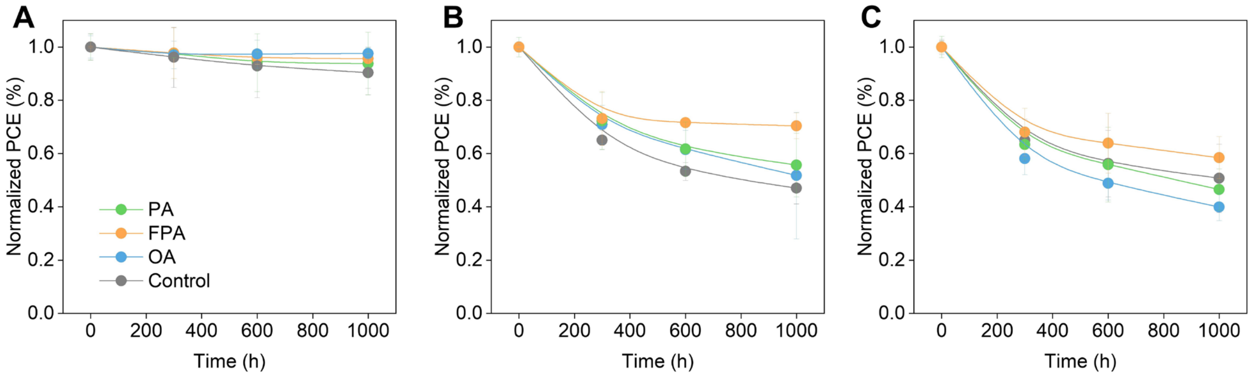

Fig. S27. Long-term stability test for control and OA-modified perovskite solar cells, under various environmental stresses. Error bars: SD, n=3. (**A**) Normalized PCE of perovskite solar cells stored in a dark atmosphere (RH=30%, T=25℃). (**B**) Normalized PCE of perovskite solar cells exposed under light illumination (1 sun) in N_2-filled glove box. (**C**) Normalized PCE of perovskite solar kept at 65 ℃ in N_2-filled glove box. All the devices in the stability test are without any encapsulation.

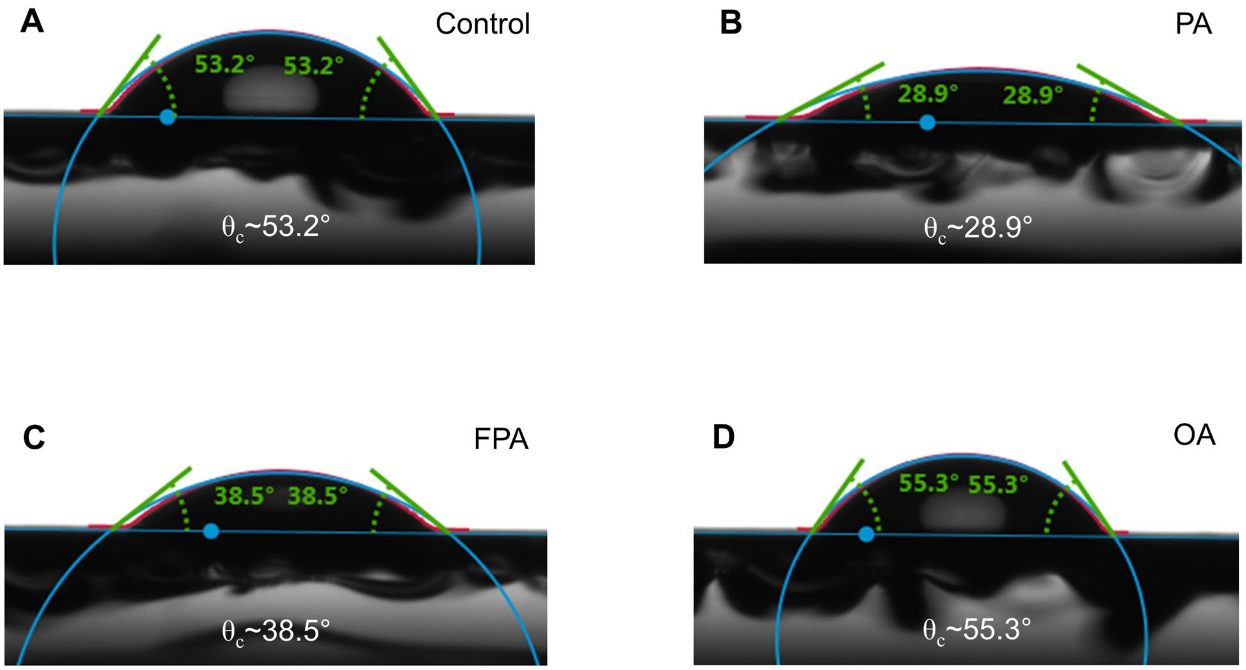

Fig. 28. Contact angle measurement of control and modified perovskites with respect to water drops.

Table S1. Bragg peak shift between modified perovskites and the control sample, derived from XRD patterns. The negative and positive sign represents (001) Bragg peak of modified samples shifting towards the left and right direction with respect to the control, respectively.

	OA	PA	FPA
5L	-0.009	-0.008	0.001
60L	-0.032	-0.025	-0.001
60H	-0.018	-0.022	-0.004

Table S2. Elliot modeling results for control and modified perovskites. The optical bandgap energy is derived from the T_{auc} plot analysis.

		OA	PA	FPA
E_x (meV)	5L	6.2	5.4	4.4
	60L	5.5	5.9	5.7
	60H	13.8	6.3	4.6
	Control	5.6		
E_g (eV)	5L	1.5984	1.5993	1.5915
	60L	1.5927	1.5987	1.5973
	60H	1.6133	1.5852	1.5883
	Control	1.5996		
Optical E_g (eV)	5L	1.5787	1.5844	1.5837
	60L	1.5752	1.5804	1.5801
	60H	1.5611	1.5720	1.5795
	Control	1.5839		

Table S3. Fitting results of TRPL for control and modified perovskites.

	β	τ_c (ns)
Control	0.5962	390.7
PA-5L	0.5080	723.8
FPA-5L	0.6395	553.3
OA-5L	0.5421	2156

Table S4. **J-V parameters for best-performing perovskite solar cells in each group, corresponding to Fig. S22 and S23.**

		V_{oc} (V)	J_{sc} (mA/cm^2)	FF (%)	PCE (%)
Control	FS	1.08	25.2	73.7	20.2
	RS	1.09	25.2	78.5	21.5
FPA-5L	FS	1.10	25.2	77.8	21.5
	RS	1.11	25.2	81.0	22.6
PA-5L	FS	1.12	25.0	80.3	22.5
	RS	1.12	25.1	82.0	23.1
OA-5L	FS	1.13	25.2	80.7	23.0
	RS	1.14	25.3	82.1	23.6
Control (LBG)	FS	1.15	25.7	79.6	23.5
	RS	1.15	25.8	80.1	23.8
OA-5L (LBG)	FS	1.18	25.9	81.9	25.0
	RS	1.18	25.9	82.1	25.2

REFERENCES AND NOTES

1. S. Sidhik, I. Metcalf, W. Li, T. Kodalle, C. J. Dolan, M. Khalili, J. Hou, F. Mandani, A. Torma, H. Zhang, R. Garai, J. Persaud, A. Marciel, I. A. Muro Puente, G. N. M. Reddy, A. Balvanz, M. A. Alam, C. Katan, E. Tsai, D. Ginger, D. P. Fenning, M. G. Kanatzidis, C. M. Sutter-Fella, J. Even, A. D. Mohite, Two-dimensional perovskite templates for durable, efficient formamidinium perovskite solar cells. *Science* **384**, 1227–1235 (2024).

2. L. Kong, Y. Sun, B. Zhao, K. Ji, J. Feng, J. Dong, Y. Wang, Z. Liu, S. Maqbool, Y. Li, Y. Yang, L. Dai, W. Lee, C. Cho, S. D. Stranks, R. H. Friend, N. Wang, N. C. Greenham, X. Yang, Fabrication of red-emitting perovskite LEDs by stabilizing their octahedral structure. *Nature* **631**, 73–79 (2024).

3. F. P. Garcia de Arquer, D. V. Talapin, V. I. Klimov, Y. Arakawa, M. Bayer, E. H. Sargent, Semiconductor quantum dots: Technological progress and future challenges. *Science* **373**, eaaz8541 (2021).

4. P. K. Nayak, S. Mahesh, H. J. Snaith, D. Cahen, Photovoltaic solar cell technologies: Analysing the state of the art. *Nat. Rev. Mater.* **4**, 269–285 (2019).

5. X. Zhao, P. Zhang, T. Liu, B. Tian, Y. Jiang, J. Zhang, Y. Tang, B. Li, M. Xue, W. Zhang, Z. Zhang, W. Guo, Operationally stable perovskite solar modules enabled by vapor-phase fluoride treatment. *Science* **385**, 433–438 (2024).

6. S. Tan, T. Huang, I. Yavuz, R. Wang, T. W. Yoon, M. Xu, Q. Xing, K. Park, D. K. Lee, C. H. Chen, R. Zheng, T. Yoon, Y. Zhao, H. C. Wang, D. Meng, J. Xue, Y. J. Song, X. Pan, N. G. Park, J. W. Lee, Y. Yang, Stability-limiting heterointerfaces of perovskite photovoltaics. *Nature* **605**, 268–273 (2022).

7. S. M. Park, M. Wei, J. Xu, H. R. Atapattu, F. T. Eickemeyer, K. Darabi, L. Grater, Y. Yang, C. Liu, S. Teale, B. Chen, H. Chen, T. Wang, L. Zeng, A. Maxwell, Z. Wang, K. R. Rao, Z. Cai, S. M. Zakeeruddin, J. T. Pham, C. M. Risko, A. Amassian, M. G. Kanatzidis, K. R. Graham, M. Gratzel, E. H. Sargent, Engineering ligand reactivity enables high-temperature operation of stable perovskite solar cells. *Science* **381**, 209–215 (2023).

8. Y. H. Lin, Vikram, F. Yang, X. L. Cao, A. Dasgupta, R. D. J. Oliver, A. M. Ulatowski, M. M. McCarthy, X. Shen, Q. Yuan, M. G. Christoforo, F. S. Y. Yeung, M. B. Johnston, N. K. Noel, L. M. Herz, M. S. Islam, H. J. Snaith, Bandgap-universal passivation enables stable perovskite solar cells with low photovoltage loss. *Science* **384**, 767–775 (2024).

9. D. Luo, R. Su, W. Zhang, Q. Gong, R. Zhu, Minimizing non-radiative recombination losses in perovskite solar cells. *Nat. Rev. Mater.* **5**, 44–60 (2020).

10. G. Kim, H. Min, K. S. Lee, D. Y. Lee, S. M. Yoon, S. I. Seok, Impact of strain relaxation on performance of α-formamidinium lead iodide perovskite solar cells. *Science* **370**, 108–112 (2020).

11. J. A. Steele, H. Jin, I. Dovgaliuk, R. F. Berger, T. Braeckevelt, H. Yuan, C. Martin, E. Solano, K. Lejaeghere, S. M. J. Rogge, C. Notebaert, W. Vandezande, K. P. F. Janssen, B. Goderis, E. Debroye, Y. K. Wang, Y. Dong, D. Ma, M. Saidaminov, H. Tan, Z. Lu, V. Dyadkin, D. Chernyshov, V. Van Speybroeck, E. H. Sargent, J. Hofkens, M. B. J. Roeffaers, Thermal unequilibrium of strained black $CsPbI_3$ thin films. *Science* **365**, 679–684 (2019).

12. Y. Chen, Y. Lei, Y. Li, Y. Yu, J. Cai, M. H. Chiu, R. Rao, Y. Gu, C. Wang, W. Choi, H. Hu, C. Wang, Y. Li, J. Song, J. Zhang, B. Qi, M. Lin, Z. Zhang, A. E. Islam, B. Maruyama, S. Dayeh, L. J. Li, K. Yang, Y. H. Lo, S. Xu, Strain engineering and epitaxial stabilization of halide perovskites. *Nature* **577**, 209–215 (2020).

13. T. A. S. Doherty, S. Nagane, D. J. Kubicki, Y. K. Jung, D. N. Johnstone, A. N. Iqbal, D. Guo, K. Frohna, M. Danaie, E. M. Tennyson, S. Macpherson, A. Abfalterer, M. Anaya, Y. H. Chiang, P. Crout, F. S. Ruggeri, S. Collins, C. P. Grey, A. Walsh, P. A. Midgley, S. D. Stranks, Stabilized tilted-octahedra halide perovskites inhibit local formation of performance-limiting phases. *Science* **374**, 1598–1605 (2021).

14. C. Zhu, X. Niu, Y. Fu, N. Li, C. Hu, Y. Chen, X. He, G. Na, P. Liu, H. Zai, Y. Ge, Y. Lu, X. Ke, Y. Bai, S. Yang, P. Chen, Y. Li, M. Sui, L. Zhang, H. Zhou, Q. Chen, Strain engineering in perovskite solar cells and its impacts on carrier dynamics. *Nat. Commun.* **10**, 815 (2019).

15. W. Meng, K. Zhang, A. Osvet, J. Zhang, W. Gruber, K. Forberich, B. Meyer, W. Heiss, T. Unruh, N. Li, C. J. Brabec, Revealing the strain-associated physical mechanisms impacting the performance and stability of perovskite solar cells. *Joule* **6**, 458–475 (2022).

16. D. Liu, D. Luo, A. N. Iqbal, K. W. P. Orr, T. A. S. Doherty, Z. H. Lu, S. D. Stranks, W. Zhang, Strain analysis and engineering in halide perovskite photovoltaics. *Nat. Mater.* **20**, 1337–1346 (2021).

17. R. Azmi, D. S. Utomo, B. Vishal, S. Zhumagali, P. Dally, A. M. Risqi, A. Prasetio, E. Ugur, F. Cao, I. F. Imran, A. A. Said, A. R. Pininti, A. S. Subbiah, E. Aydin, C. Xiao, S. I. Seok, S. De Wolf, Double-side 2D/3D heterojunctions for inverted perovskite solar cells. *Nature* **628**, 93–98 (2024).

18. F. Zhang, S. Y. Park, C. Yao, H. Lu, S. P. Dunfield, C. Xiao, S. Ulicna, X. Zhao, L. Du Hill, X. Chen, X. Wang, L. E. Mundt, K. H. Stone, L. T. Schelhas, G. Teeter, S. Parkin, E. L. Ratcliff, Y. L. Loo, J. J. Berry, M. C. Beard, Y. Yan, B. W. Larson, K. Zhu, Metastable Dion-Jacobson 2D structure enables efficient and stable perovskite solar cells. *Science* **375**, 71–76 (2022).

19. D. Luo, W. Yang, Z. Wang, A. Sadhanala, Q. Hu, R. Su, R. Shivanna, G. F. Trindade, J. F. Watts, Z. Xu, T. Liu, K. Chen, F. Ye, P. Wu, L. Zhao, J. Wu, Y. Tu, Y. Zhang, X. Yang, W. Zhang, R. H. Friend, Q. Gong, H. J. Snaith, R. Zhu, Enhanced photovoltage for inverted planar heterojunction perovskite solar cells. *Science* **360**, 1442–1446 (2018).

20. R. Lin, Y. Wang, Q. Lu, B. Tang, J. Li, H. Gao, Y. Gao, H. Li, C. Ding, J. Wen, P. Wu, C. Liu, S. Zhao, K. Xiao, Z. Liu, C. Ma, Y. Deng, L. Li, F. Fan, H. Tan, All-perovskite tandem solar cells with 3D/3D bilayer perovskite heterojunction. *Nature* **620**, 994–1000 (2023).

21. Z. Chen, Q. Cheng, H. Chen, Y. Wu, J. Ding, X. Wu, H. Yang, H. Liu, W. Chen, X. Tang, X. Lu, Y. Li, Y. Li, Perovskite grain-boundary manipulation using room-temperature dynamic self-healing “ligaments” for developing highly stable flexible perovskite solar cells with 23.8% efficiency. *Adv. Mater.* **35**, e2300513 (2023).

22. N. Yang, C. Zhu, Y. Chen, H. Zai, C. Wang, X. Wang, H. Wang, S. Ma, Z. Gao, X. Wang, J. Hong, Y. Bai, H. Zhou, B.-B. Cui, Q. Chen, An in situ cross-linked 1D/3D perovskite heterostructure improves the stability of hybrid perovskite solar cells for over 3000 h operation. *Energ. Environ. Sci.* **13**, 4344–4352 (2020).

23. X. Li, Z. Ying, J. Zheng, X. Wang, Y. Chen, M. Wu, C. Xiao, J. Sun, C. Shou, Z. Yang, Y. Zeng, X. Yang, J. Ye, Surface reconstruction for efficient and stable monolithic perovskite/silicon tandem solar cells with greatly suppressed residual strain. *Adv. Mater.* **35**, e2211962 (2023).

24. H. Wang, C. Zhu, L. Liu, S. Ma, P. Liu, J. Wu, C. Shi, Q. Du, Y. Hao, S. Xiang, H. Chen, P. Chen, Y. Bai, H. Zhou, Y. Li, Q. Chen, Interfacial residual stress relaxation in perovskite solar cells with improved stability. *Adv. Mater.* **31**, e1904408 (2019).

25. N. Li, S. Apergi, C. C. S. Chan, Y. Jia, F. Xie, Q. Liang, G. Li, K. S. Wong, G. Brocks, S. Tao, N. Zhao, Diammonium-mediated perovskite film formation for high-luminescence red perovskite light-emitting diodes. *Adv. Mater.* **34**, e2202042 (2022).

26. B. Chen, S. R. Wang, X. Zhang, W. K. Zhu, Z. Y. Cao, F. Hao, Reducing the interfacial voltage loss in tin halides perovskite solar cells. *Chem. Eng. J.* **445**, 136769 (2022).

27. K. Zhang, A. Vincze, E. Metwalli, J. Zhang, C. Liu, W. Meng, B. Zhang, J. Tian, T. Heumueller, Z. Xie, J. Luo, A. Osvet, T. Unruh, L. Lüer, N. Li, C. J. Brabec, Impact of 2D ligands on lattice strain and energy losses in narrow-bandgap lead–Tin perovskite solar cells. *Adv. Funct. Mater.* **33**, 2303455 (2023).

28. L. A. Muscarella, B. Ehrler, The influence of strain on phase stability in mixed-halide perovskites. *Joule* **6**, 2016–2031 (2022).

29. J. Zhao, Y. Deng, H. Wei, X. Zheng, Z. Yu, Y. Shao, J. E. Shield, J. Huang, Strained hybrid perovskite thin films and their impact on the intrinsic stability of perovskite solar cells. *Sci. Adv.* **3**, eaao5616 (2017).

30. D. J. Xue, Y. Hou, S. C. Liu, M. Wei, B. Chen, Z. Huang, Z. Li, B. Sun, A. H. Proppe, Y. Dong, M. I. Saidaminov, S. O. Kelley, J. S. Hu, E. H. Sargent, Regulating strain in perovskite thin films through charge-transport layers. *Nat. Commun.* **11**, 1514 (2020).

31. N. Rolston, K. A. Bush, A. D. Printz, A. Gold-Parker, Y. Ding, M. F. Toney, M. D. McGehee, R. H. Dauskardt, Engineering stress in perovskite solar cells to improve stability. *Adv. Energy Mater.* **8**, 1802139 (2018).

32. T. Du, T. J. Macdonald, R. X. Yang, M. Li, Z. Jiang, L. Mohan, W. Xu, Z. Su, X. Gao, R. Whiteley, C. T. Lin, G. Min, S. A. Haque, J. R. Durrant, K. A. Persson, M. A. McLachlan, J. Briscoe, Additive-free, low-temperature crystallization of stable α-$FAPbI_3$ perovskite. *Adv. Mater.* **34**, e2107850 (2022).

33. R. Chen, J. A. Wang, Z. H. Liu, F. M. Ren, S. W. Liu, J. Zhou, H. X. Wang, X. Meng, Z. Zhang, X. Y. Guan, W. X. Liang, P. A. Troshin, Y. B. Qi, L. Y. Han, W. Chen, Reduction of bulk and surface defects in inverted methylammonium- and bromide-free formamidinium perovskite solar cells. *Nat. Energy* **8**, 839–849 (2023).

34. M. Wang, Z. Shi, C. Fei, Z. J. D. Deng, G. Yang, S. P. Dunfield, D. P. Fenning, J. Huang, Ammonium cations with high pKa in perovskite solar cells for improved high-temperature photostability. *Nat. Energy* **8**, 1229–1239 (2023).

35. L. Luo, H. P. Zeng, Z. W. Wang, M. Li, S. You, B. Chen, A. Maxwell, Q. Y. An, L. M. Cui, D. Y. Luo, J. T. Hu, S. Z. Li, X. Q. Cai, W. X. Li, L. Li, R. Guo, R. Huang, W. X. Liang, Z. H. Lu, L. Q. Mai, Y. G. Rong, E. H. Sargent, X. Li, Stabilization of 3D/2D perovskite heterostructures via inhibition of ion diffusion by cross-linked polymers for solar cells with improved performance. *Nat. Energy* **8**, 294–303 (2023).

36. D. W. deQuilettes, S. M. Vorpahl, S. D. Stranks, H. Nagaoka, G. E. Eperon, M. E. Ziffer, H. J. Snaith, D. S. Ginger, Impact of microstructure on local carrier lifetime in perovskite solar cells. *Science* **348**, 683–686 (2015).

37. Z. Yang, J. Lu, M. ZhuGe, Y. Cheng, J. Hu, F. Li, S. Qiao, Y. Zhang, G. Hu, Q. Yang, D. Peng, K. Liu, C. Pan, Controllable growth of aligned monocrystalline $CsPbBr_3$ microwire arrays for piezoelectric-induced dynamic modulation of single-mode lasing. *Adv. Mater.* **31**, e1900647 (2019).

38. K. Leng, W. Fu, Y. Liu, M. Chhowalla, K. P. Loh, From bulk to molecularly thin hybrid perovskites. *Nat. Rev. Mater.* **5**, 482–500 (2020).

39. X. Chen, H. Lu, Y. Yang, M. C. Beard, Excitonic effects in methylammonium lead halide perovskites. *J. Phys. Chem. Lett.* **9**, 2595–2603 (2018).

40. B. Guo, C. Luo, C. Yan, B. Sun, W. Li, W. Yang, Understanding excitonic behavior in light absorption and recombination process. *J. Phys. Chem. C* **124**, 26076–26082 (2020).

41. S. Singh, C. Li, F. Panzer, K. L. Narasimhan, A. Graeser, T. P. Gujar, A. Kohler, M. Thelakkat, S. Huettner, D. Kabra, Effect of thermal and structural disorder on the electronic structure of hybrid perovskite semiconductor $CH_3NH_3PbI_3$. *J. Phys. Chem. Lett.* **7**, 3014–3021 (2016).

42. P. S. Prevéy, X-ray diffraction residual stress techniques, in *ASM Handbook* (1986), vol. 10, pp. 380–392.

43. N.-G. Park, K. Zhu, Scalable fabrication and coating methods for perovskite solar cells and solar modules. *Nat. Rev. Mater.* **5**, 333–350 (2020).

44. D. G. Neerinck, T. J. Vink, Depth profiling of thin ITO films by grazing incidence X-ray diffraction. *Thin Solid Films* **278**, 12–17 (1996).

45. P. Staron, A. Schreyer, H. Clemens, S. Mayer, *Neutrons and synchrotron radiation in engineering materials science: From fundamentals to applications*. (John Wiley & Sons, 2017).

46. Z. J. Peng, A. Vincze, F. Streller, V. M. Le Corre, K. C. Zhang, C. H. Li, J. J. Tian, C. Liu, J. S. Luo, Y. C. Zhao, A. Späth, R. Fink, T. Heumüller, A. Osvet, N. Li, M. Stolterfoht, L. Lüer, C. J. Brabec, Revealing degradation mechanisms in 3D/2D perovskite solar cells under photothermal accelerated ageing. *Energ. Environ. Sci.* **17**, 8313–8324 (2024).

47. C. Zhang, S. Wu, L. Tao, G. M. Arumugam, C. Liu, Z. Wang, S. Zhu, Y. Yang, J. Lin, X. Liu, R. E. I. Schropp, Y. Mai, Fabrication strategy for efficient 2D/3D perovskite solar cells enabled by diffusion passivation and strain compensation. *Adv. Energy Mater.* **10**, 2002004 (2020).

48. G. Yang, Z. W. Ren, K. Liu, M. C. Qin, W. Y. Deng, H. K. Zhang, H. B. Wang, J. W. Liang, F. H. Ye, Q. Liang, H. Yin, Y. X. Chen, Y. L. Zhuang, S. Q. Li, B. W. Gao, J. B. Wang, T. T. Shi, X. Wang, X. H. Lu, H. B. Wu, J. H. Hou, D. Y. Lei, S. K. So, Y. Yang, G. J. Fang, G. Li, Stable and low-photovoltage-loss perovskite solar cells by multifunctional passivation. *Nat. Photonics* **15**, 681–689 (2021).

49. R. Azmi, E. Ugur, A. Seitkhan, F. Aljamaan, A. S. Subbiah, J. Liu, G. T. Harrison, M. I. Nugraha, M. K. Eswaran, M. Babics, Y. Chen, F. Xu, T. G. Allen, A. U. Rehman, C. L. Wang, T. D. Anthopoulos, U. Schwingenschlogl, M. De Bastiani, E. Aydin, S. De Wolf, Damp heat-stable perovskite solar cells with tailored-dimensionality 2D/3D heterojunctions. *Science* **376**, 73–77 (2022).

50. Q. Tu, I. Spanopoulos, S. Hao, C. Wolverton, M. G. Kanatzidis, G. S. Shekhawat, V. P. Dravid, Out-of-plane mechanical properties of 2D hybrid organic-inorganic perovskites by nanoindentation. *ACS Appl. Mater. Interfaces* **10**, 22167–22173 (2018).

51. W. D. Callister, *Materials Science and Engineering: An Introduction* (John Wiley & Sons Incorporated, ed. 7, 2006).

52. J. C. Blancon, J. Even, C. C. Stoumpos, M. G. Kanatzidis, A. D. Mohite, Semiconductor physics of organic-inorganic 2D halide perovskites. *Nat. Nanotechnol.* **15**, 969–985 (2020).

53. H. Kum, D. Lee, W. Kong, H. Kim, Y. Park, Y. Kim, Y. Baek, S. H. Bae, K. Lee, J. Kim, Epitaxial growth and layer-transfer techniques for heterogeneous integration of materials for electronic and photonic devices. *Nat. Electron.* **2**, 439–450 (2019).

54. Q. Zhou, J. Duan, X. Yang, Y. Duan, Q. Tang, Interfacial strain release from the $WS_2/CsPbBr_3$ van der waals heterostructure for 1.7 V voltage all-inorganic perovskite solar cells. *Angew. Chem. Int. Ed. Engl.* **59**, 21997–22001 (2020).

55. J. T. Cremer, in *Advances in Imaging and Electron Physics*, J. T. Cremer, Ed. (Elsevier, 2012), vol. 173, pp. 349–415.

56. A. Bonadio, F. P. Sabino, A. L. M. Freitas, M. R. Felez, G. M. Dalpian, J. A. Souza, Comparing the cubic and tetragonal phases of $MAPbI_3$ at room Temperature. *Inorg. Chem.* **62**, 7533–7544 (2023).

57. A. Ren, H. Wang, L. Dai, J. Xia, X. Bai, E. Butler-Caddle, J. A. Smith, H. Lai, J. Ye, X. Li, S. Zhan, C. Yao, Z. Li, M. Tang, X. Liu, J. Bi, B. Li, S. Kai, R. Chen, H. Yan, J. Hong, L. Yuan, I. P. Marko, A. Wonfor, F. Fu, S. A. Hindmarsh, A. M. Sanchez, J. Lloyd-Hughes, S. J. Sweeney, A. Rao, N. C. Greenham, J. Wu, Y. Li, Q. Cheng, R. H. Friend, R. V. Penty, I. H. White, H. J. Snaith, W. Zhang, High-bandwidth perovskite photonic sources on silicon. *Nat. Photonics* **17**, 798–805 (2023).

58. M. Baranowski, P. Plochocka, Excitons in metal-halide perovskites. *Adv. Energy Mater.* **10**, 1903659(2020).

59. J. Fu, J. Zhang, T. Zhang, L. Yuan, Z. Zhang, Z. Jiang, Z. Huang, T. Wu, K. Yan, L. Zhang, A. Wang, W. Ji, Y. Zhou, B. Song, Synergistic effects of interfacial energy level regulation and stress relaxation via a buried interface for highly efficient perovskite solar cells. *ACS Nano* **17**, 2802–2812 (2023).

60. X. K. Liu, W. Xu, S. Bai, Y. Jin, J. Wang, R. H. Friend, F. Gao, Metal halide perovskites for light-emitting diodes. *Nat. Mater.* **20**, 10–21 (2021).

61. G. F. Harrington, J. Santiso, Back-to-basics tutorial: X-ray diffraction of thin films. *J. Electroceram.* **47**, 141–163 (2021).

62. J. T.-W. Wang, Z. Wang, S. Pathak, W. Zhang, D. W. deQuilettes, F. Wisnivesky-Rocca-Rivarola, J. Huang, P. K. Nayak, J. B. Patel, H. A. Mohd Yusof, Y. Vaynzof, R. Zhu, I. Ramirez, J. Zhang, C. Ducati, C. Grovenor, M. B. Johnston, D. S. Ginger, R. J. Nicholas, H. J. Snaith, Efficient perovskite solar cells by metal ion doping. *Energ. Environ. Sci.* **9**, 2892–2901 (2016).

63. Q. Xiong, X. F. Huang, C. Wang, Q. Zhou, Y. Gang, T. H. Li, C. Z. Hu, N. Zhang, X. B. Wang, J. H. Wu, Z. H. Su, X. Y. Gao, X. Li, N. F. Zheng, P. Gao, Managed spatial strain uniformity for efficient perovskite photovoltaics enables minimized energy deficit. *Joule* **8**, 817–834 (2024).

64. H. Chen, C. Liu, J. Xu, A. Maxwell, W. Zhou, Y. Yang, Q. Zhou, A. S. R. Bati, H. Wan, Z. Wang, L. Zeng, J. Wang, P. Serles, Y. Liu, S. Teale, Y. Liu, M. I. Saidaminov, M. Li, N. Rolston, S. Hoogland, T. Filleter, M. G. Kanatzidis, B. Chen, Z. Ning, E. H. Sargent, Improved charge extraction in inverted perovskite solar cells with dual-site-binding ligands. *Science* **384**, 189–193 (2024).

65. S. Jariwala, H. Y. Sun, G. W. P. Adhyaksa, A. Lof, L. A. Muscarella, B. Ehrler, E. C. Garnett, D. S. Ginger, Local crystal misorientation influences non-radiative recombination in halide perovskites. *Joule* **3**, 3048–3060 (2019).

66. K. W. P. Orr, J. Diao, K. Dey, M. Hameed, M. Dubajic, H. L. Gilbert, T. A. Selby, S. J. Zelewski, Y. Han, M. R. Fitzsimmons, B. Roose, P. Li, J. Fan, H. Jiang, J. Briscoe, I. K. Robinson, S. D. Stranks, Strain heterogeneity and extended defects in halide perovskite devices. *ACS Energy Lett.* **9**, 3001–3011 (2024).

67. J. Euvrard, Y. F. Yan, D. B. Mitzi, Electrical doping in halide perovskites. *Nat. Rev. Mater.* **6**, 531–549 (2021).

68. X. Jiang, F. Wang, Q. Wei, H. Li, Y. Shang, W. Zhou, C. Wang, P. Cheng, Q. Chen, L. Chen, Z. Ning, Ultra-high open-circuit voltage of tin perovskite solar cells via an electron transporting layer design. *Nat. Commun.* **11**, 1245 (2020).

69. D. A. Neamen, *Semiconductor physics and devices* (McGraw-Hill Education, 2011).

70. T. Xu, Q. Qiao, Conjugated polymer–Inorganic semiconductor hybrid solar cells. *Energ. Environ. Sci.* **4**, 2700–2720 (2011).

71. J. M. Ball, A. Petrozza, Defects in perovskite-halides and their effects in solar cells. *Nat. Energy* **1**, 1–13 (2016).

72. H. Min, D. Y. Lee, J. Kim, G. Kim, K. S. Lee, J. Kim, M. J. Paik, Y. K. Kim, K. S. Kim, M. G. Kim, T. J. Shin, S. Il Seok, Perovskite solar cells with atomically coherent interlayers on SnO_2 electrodes. *Nature* **598**, 444–450 (2021).

73. J. Zhang, GIXSGUI: A MATLAB toolbox for grazing-incidence X-ray scattering data visualization and reduction, and indexing of buried three-dimensional periodic nanostructured films. *J. Appl. Cryst.* **48**, 917–926 (2015).

74. N. Zhao, C. Yang, F. Bian, D. Guo, X. Ouyang, SGTools: A suite of tools for processing and analyzing large data sets from in situ X-ray scattering experiments. *J. Appl. Cryst.* **55**, 195–203 (2022).

75. T. Lu, F. Chen, Multiwfn: A multifunctional wavefunction analyzer. *J. Comput. Chem.* **33**, 580–592 (2012).

76. P. Shi, J. Xu, I. Yavuz, T. Huang, S. Tan, K. Zhao, X. Zhang, Y. Tian, S. Wang, W. Fan, Y. Li, D. Jin, X. Yu, C. Wang, X. Gao, Z. Chen, E. Shi, X. Chen, D. Yang, J. Xue, Y. Yang, R. Wang, Strain regulates the photovoltaic performance of thick-film perovskites. *Nat. Commun.* **15**, 2579 (2024).

77. S. K. Yadavalli, Z. Dai, H. Zhou, Y. Zhou, N. P. Padture, Facile healing of cracks in organic–inorganic halide perovskite thin films. *Acta Mater.* **187**, 112–121 (2020).

78. C. Ramirez, S. K. Yadavalli, H. F. Garces, Y. Zhou, N. P. Padture, Thermo-mechanical behavior of organic-inorganic halide perovskites for solar cells. *Scr. Mater.* **150**, 36–41 (2018).

79. L. B. Freund, S. Suresh, *Thin film materials: Stress, defect formation and surface evolution*. (Cambridge Univ. Press, 2010).

80. C. C. Stoumpos, C. D. Malliakas, M. G. Kanatzidis, Semiconducting tin and lead iodide perovskites with organic cations: Phase transitions, high mobilities, and near-infrared photoluminescent properties. *Inorg. Chem.* **52**, 9019–9038 (2013).

81. S. Kavadiya, J. Strzalka, D. M. Niedzwiedzki, P. Biswas, Crystal reorientation in methylammonium lead iodide perovskite thin film with thermal annealing. *J. Mater. Chem. A* **7**, 12790–12799 (2019).

82. M. Jung, T. J. Shin, J. Seo, G. Kim, S. I. Seok, Structural features and their functions in surfactant-armoured methylammonium lead iodide perovskites for highly efficient and stable solar cells. *Energ. Environ. Sci.* **11**, 2188–2197 (2018).

83. S.-i. Fukada, H. Yamamoto, R. Ikeda, D. Nakamura, Hydrogen-1 nuclear magnetic resonance, differential thermal analysis, X-ray powder diffraction and electrical conductivity studies on the motion of cations, including self-diffusion in crystals of propylammonium chloride and bromide as well as their n-deuterated analogues. *J. Chem. Soc. Faraday Trans. 1* **83**, 3207–3222 (1987).
